# Epidermal growth factor can signal via β-catenin to control proliferation of mesenchymal stem cells independently of canonical Wnt signalling

**DOI:** 10.1016/j.cellsig.2018.09.021

**Published:** 2019-01

**Authors:** Charlotte Knight, Sally James, David Kuntin, James Fox, Katherine Newling, Sam Hollings, Rebecca Pennock, Paul Genever

**Affiliations:** Department of Biology, University of York, York YO10 5DD, United Kingdom

**Keywords:** Epidermal growth factor, β-Catenin, Mesenchymal stem cells, Integrin-linked kinase

## Abstract

Bone marrow mesenchymal stem/stromal cells (MSCs) maintain bone homeostasis and repair through the ability to expand in response to mitotic stimuli and differentiate into skeletal lineages. Signalling mechanisms that enable precise control of MSC function remain unclear. Here we report that by initially examining differences in signalling pathway expression profiles of individual MSC clones, we identified a previously unrecognised signalling mechanism regulated by epidermal growth factor (EGF) in primary human MSCs. We demonstrate that EGF is able to activate β-catenin, a key component of the canonical Wnt signalling pathway. EGF is able to induce nuclear translocation of β-catenin in human MSCs but does not drive expression of Wnt target genes or T cell factor (TCF) activity in MSC reporter cell lines. Using an efficient Design of Experiments (DoE) statistical analysis, with different combinations and concentrations of EGF and Wnt ligands, we were able to confirm that EGF does not influence the Wnt/β-catenin pathway in MSCs. We show that the effects of EGF on MSCs are temporally regulated to initiate early “classical” EGF signalling mechanisms (e.g via mitogen activated protein kinase) with delayed activation of β-catenin. By RNA-sequencing, we identified gene sets that were exclusively regulated by the EGF/β-catenin pathway, which were distinct from classical EGF-regulated genes. However, subsets of classical EGF gene targets were significantly influenced by EGF/β-catenin activation. These signalling pathways cooperate to enable EGF-mediated proliferation of MSCs by alleviating the suppression of cell cycle pathways induced by classical EGF signalling.

## Highlights

• Epidermal growth factor (EGF) controls mesenchymal stem cell (MSC) proliferation.
• EGF signals through β-catenin in MSCs but not in related fibroblastic cells.
• Classical EGF and EGF/β-catenin cooperatively regulate distinct gene sets in MSCs.
• EGF/β-catenin enables MSC proliferation by alleviating cell cycle suppression.

## 1 Introduction

Mesenchymal stem/stromal cells (MSCs, also referred to as bone marrow stromal cells or mesenchymal stem cells) are a major stromal component of bone marrow. Related cell types are present in many other tissues, where they often act to support parenchymal cell function [1,2]. MSCs have the capacity to form fibroblastic colony-forming units (CFU-f), due to the presence of a progenitor cell population and are able to differentiate into osteoblasts, chondrocytes and adipocytes [3], however MSC cultures are heterogeneous with no single selective and widely adopted MSC surface marker [4,5]. Despite intensive efforts, there is still a distinct lack of understanding of the specific mechanisms regulating MSC proliferation and differentiation, which may in part be due to the heterogeneity of the MSC pool. In our own studies of immortalised clonal MSC lines [6], we found significant variations in potency, cell morphology and global expression profiles with particularly striking differences in genes involved in Wnt, receptor tyrosine kinase (RTK) growth factor signalling and integrin-linked kinase (ILK)-regulated pathways. These signalling cascades have wide-ranging effects on development and tissue homeostasis through the regulation of essential cellular processes including survival, proliferation, migration, stem cell maintenance and differentiation.

Canonical Wnt signalling involves the regulatory complex composed of adenomatous polyposis coli (APC)/Axin/GSK-3β [7]. In the presence of Wnt ligands bound to Frizzled receptors and LRP5/6 co-receptors, the intracellular protein Dishevelled becomes activated and displaces GSK-3β from the APC/Axin complex, suppressing GSK-3β-mediated phosphorylation of β-catenin, thereby preventing its ubiquitination and proteasomal degradation. This results in the nuclear translocation of dephosphorylated active β-catenin and recruitment of T cell factor/lymphoid enhancer factor (TCF/LEF) binding factors as co-activators for activation of gene transcription [8,9]. Wnt/β-catenin signalling is associated with the regulation of bone mass. A reduction in canonical Wnt signalling is associated with low bone mineral density in patients with osteoporosis pseudoglioma syndrome that exhibit a loss of function mutation in LRP5 [10] and increased bone is observed in activating LRP5 mutations [11]. Wnt signalling is also important in the control of MSC function and osteogenic induction, though with variable interpretations [[11], [12], [13], [14], [15], [16]].

**Fig. 1:**
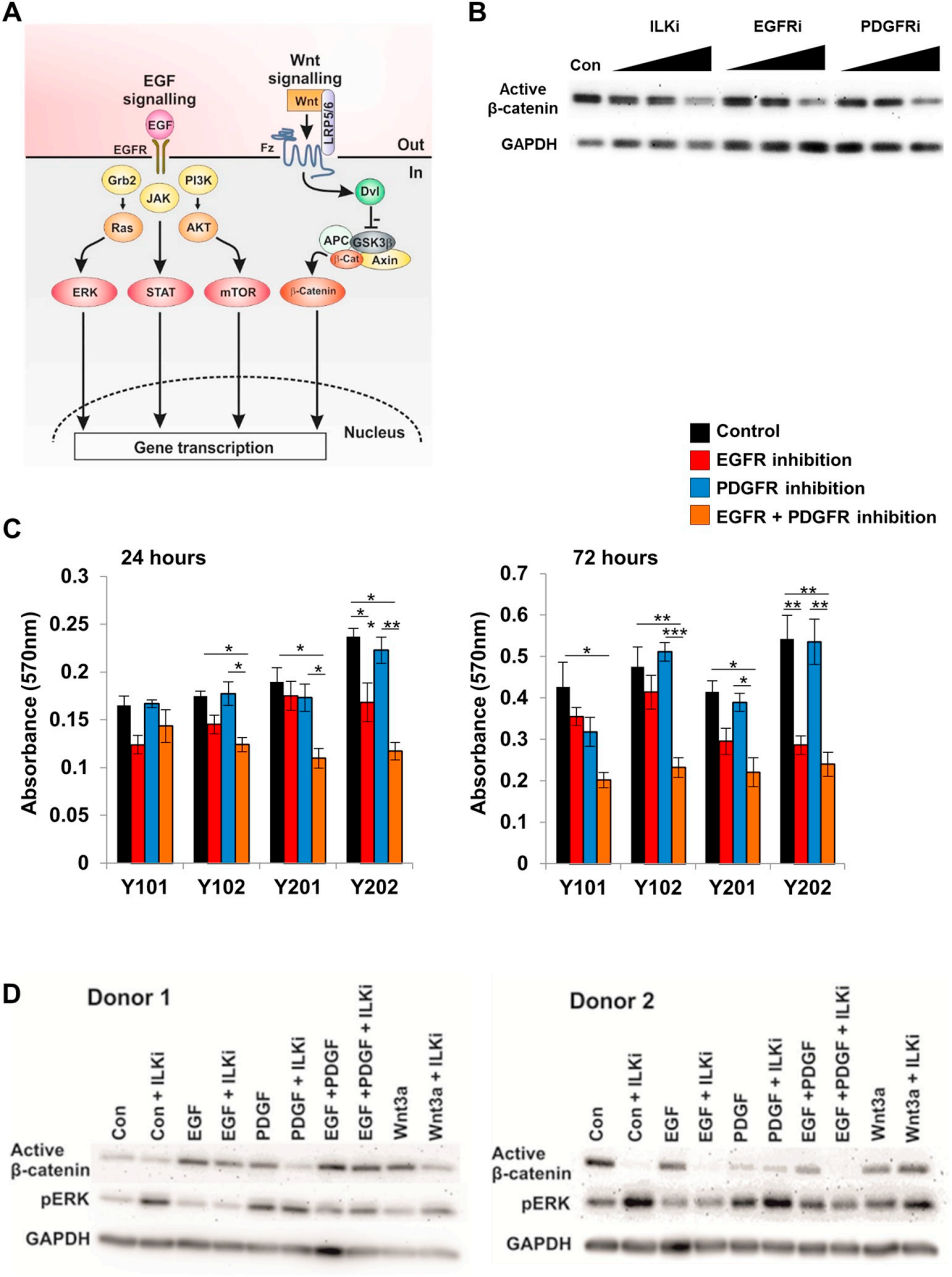
RTK, ILK and β-catenin signalling are interrelated in human MSCs. (A) Schematic of EGF and Wnt/β-catenin signal transduction mechanisms. (B) Analysis of the effects of ILK, EGFR and PDGFR signalling inhibition on active β-catenin levels in Y202 hTERT MSCs by western blotting. (C) MTT assay of viable cell numbers of hTERT MSCs following 24 and 72 h of RTK signalling inhibition, graphs show mean absorbance ± SEM (*n* = 6), * = *p* < .05, ** = *p* < .005, *** = *p* < .001. (D) Western blot analysis of active β-catenin and pERK levels in primary MSCs following EGF and PDGF exposure in the presence and absence of ILK inhibition (ILKi), donors K102 and K96 shown. Densitometric analysis of band intensity normalised to GAPDH expression is shown in Fig. S1B. See also Fig. S1.

Although GSK3β and β-catenin are key components of the canonical Wnt signalling cascade, their biological functions are not exclusive to this pathway, and they act as points of crosstalk between Wnt/β-catenin and other key cellular signalling mechanisms. For example, integrin-linked kinase (ILK) is a serine/threonine kinase and downstream effector of integrin and growth factor signalling [17] that enhances β-catenin signalling through the phosphorylation and inactivation of GSK3β [18,19]. RTK signalling pathways, including epidermal growth factor (EGF), platelet-derived growth factor (PDGF) and fibroblast growth factor (FGF), can also influence β-catenin function. PDGF treatment is able to drive nuclear translocation of β-catenin by interfering with its interaction with Axin and GSK3β and the subsequent inhibition of β-catenin phosphorylation [20]. EGF-induced LRP6 phosphorylation by the extracellular-signal-regulated kinase/mitogen activated protein kinase (ERK/MAPK) pathway has been shown to activate β-catenin signalling [21,22] and FGF signalling through MAPK can stimulate bone formation by preventing β-catenin degradation, mediated by the recruitment of a deubiquitinating enzyme [23]. EGF also has widespread effects on bone and cartilage development [[24], [25], [26], [27]] and can influence the proliferation and differentiation of MSCs [[28], [29], [30], [31], [32]], though sometimes with apparently conflicting data. This may be related to specific effects of EGF on MSC proliferation versus differentiation and/or an incomplete understanding of interconnected downstream signal transduction pathways as the vast majority of studies consider EGF- and β-catenin-mediated signalling independently (Fig. 1A). Here we demonstrate for the first time that β-catenin can be activated via a Wnt ligand-independent mechanism in human MSCs, through EGF, which runs parallel to, but is distinct from the canonical Wnt/β-catenin pathway. The EGF/β-catenin and classical EGF signalling pathways regulate discrete gene sets that act in concert to control EGF-mediated proliferation of MSCs.

## 2 Methods

### 2.1 Cell culture

In this study, we used human clonal MSC lines, immortalised with human telomerase reverse transcriptase (hTERT-MSCs) and primary human MSC cultures. hTERT-MSCs were used initially as differences in EGF-mediated signalling were first identified in comparative analyses of the different MSC lines. Primary MSCs from different donors were used to ensure data were not an artefact of the immortalisation process, donor ID codes are provided in the figure legends. Bone samples were obtained with written informed consent from Clifton Park NHS Treatment Centre, under approval of the Local Research Ethics Committee. MSCs were extracted from bone samples as previously described [13]. MSCs were maintained in Dulbecco's Modified Eagle's Medium (DMEM, Invitrogen) containing 1% penicillin/streptomycin (P/S, Invitrogen) and 15% foetal bovine serum (FBS, Biosera), referred to as MSC basal medium. In all experiments, MSCs were used up to passage 5. hTERT-MSCs were previously generated in the laboratory [6] and maintained in DMEM as described above but with 10% FBS, as were human dermal fibroblasts (HDFs, Fisher Scientific, Cat. No.10407693).

### 2.2 Western blot analysis

Cell cultures were lysed in RIPA buffer (Thermo Scientific) containing 0.5% Protease Inhibitor III (Merck) and 1 mM Sodium Orthovanadate (Sigma). Samples were electrophoresed and then transferred to a PVDF membrane. Membranes were probed with primary antibodies overnight at 4 °C, followed by HRP-conjugated secondary antibodies. Antibody information is provided in the Supplemental Material. Protein was visualised using enhanced chemiluminescence reagents (ECL, GE Healthcare), according to manufacturer's instructions and quantification was performed using Image J. For western blot analysis following treatment with type I collagen, tissue culture plastic was coated with type I collagen (Sigma, 3, 6 and 10 mg/ml) and then pre-incubated in MSC basal medium overnight. Cells were seeded at a density of 2.1 × 10^4^ per cm^2^ and incubated for 18 h before analysis by Western Blotting.

### 2.3 Inhibitor studies

Cells were seeded at a density of 2.1 × 10^4^ cells per cm^2^ in the appropriate basal medium for all inhibitor studies described in this section, experiments began following overnight adherence. hTERT-MSCs were treated with an ILK inhibitor, ILKi (Cpd-22; 0.6, 1, 2.5 μM; Merck), EGFR inhibitor, EGFRi (PD153035; 0.6, 1, 1.4 μM; Santa Cruz) or PDGFR inhibitor, PDGFRi (sc-222142; 0.3, 0.5, 0.8 μM; Santa Cruz) for 24 h. Primary MSCs and HDFs were incubated in DMEM supplemented with 1% P/S and 0.5% FBS (synchronisation medium) for 24 h, before a further incubation (15 min or 6 h) with recombinant EGF protein (1-100 nM, Cell Guidance Systems). For RGD studies, primary MSCs were incubated in synchronisation medium for 24 h. Samples were incubated overnight with 1 μM ILKi or vehicle control after which RGD integrin binding peptide (100 μg/ml, Anaspec) was added to samples for 6 h. For growth factor plus ILKi studies, primary MSCs were incubated overnight with 1 μM ILKi or vehicle control before a further incubation period of 24 h untreated, or treated with 1 μM ILKi, 10 nM recombinant EGF, PDGF (20 nM, Cell Guidance Systems) alone or in combination.

### 2.4 MTT assays of viable cell number

Cells were seeded at a density of 3.6 × 10^3^ per cm^2^ and left overnight to adhere. hTERT-MSCs were then incubated with either vehicle control, 1Μm EGFRi or 0.5 μM PDGFRi, alone or in combination for a period of 24 or 72 h. Primary MSCs were incubated overnight with 1 μM ILKi or vehicle control treated before the addition of 10 nM EGF for 24 or 48 h. For samples exposed to the AP-1 inhibitor, treatment began after overnight adherence. Samples were treated with 10 nM EGF alone or in combination with the AP-1 inhibitor (17–68 nM, Biotechne, Cat. No. 2476) and were incubated for up to 120 h. Similarly, primary MSCs were treated with the Wnt pathway inhibitor (5 or 10 μM IWR-1, Sigma, Cat. No. I0161) with or without 10 nM EGF. 5 mg/ml MTT reagent was then added to the above samples and incubated at 37 °C for 3 h. Cells were lysed in 0.1 M acidic HCl in absolute isopropanol before absorbance was read at 570 nm.

### 2.5 Flow cytometry

MSCs were detached from tissue culture plastic with wash buffer (0.2% BSA and 5 mM EDTA in 1× PBS), before incubation with primary antibodies: PDGFRα (0.5 μg/ml per 1 × 10^6^ cells, Santa Cruz,) and EGFR (1 μg/ml per 1 × 10^6^ cells, Abcam). Samples were then incubated with FITC conjugated secondary antibodies (Thermo Fisher Scientific) followed by incubation with DAPI. Flow cytometric analysis was performed using a CyAn ADP flow cytometer and analysed using Summit software (v4.3, Beckman Coulter).

### 2.6 Immunofluorescence

Primary MSCs were seeded at 2 × 10^4^ cells per cm^2^ onto glass coverslips and left to adhere overnight. Cells were treated with varying concentrations of EGF (1 nM, 10 nM and 100 nM) and left for 24 h. Samples were fixed for 15 min in 4% paraformaldehyde before incubation with anti-active β-catenin (1:200, Merck) for 1 h at room temperature. Secondary antibody (Alexor Fluor 647, Invitrogen) was added for a further hour in the dark at room temperature. Samples were counterstained with DAPI, and mounted using Prolong Gold Antifade mountant before visualisation using the LSM710 confocal microscopy system.

### 2.7 Quantitative real time polymerase chain reaction (QPCR)

Cell cultures were lysed in 0.5 ml Trizol (Life Technologies) for 5 min, and frozen at -80 °C. RNA was then extracted by chloroform phase separation and purification. cDNA was synthesised from 1 μg RNA. QPCR was performed on the Applied Biosystems 7300 Real Time PCR System using SYBR Green PCR Master Mix. Primer sequences are given in the Supplemental Material. Fold changes are shown as 2^(−ΔΔCt)^, normalised to expression of RPS27a and relative to expression in untreated controls.

### 2.8 TCF reporter assays

Y201 hTERT-MSCs were lentivirally transduced with a TCF-EGFP reporter (Qiagen) to generate a stable MSC-Wnt-EGFP reporter line as previously described [33], and treated with recombinant EGF or Wnt3a. After 24 h of treatment, cells were visualised by fluorescence microscopy or harvested in wash buffer for flow cytometric analysis (see above). The mouse mesenchymal cell line, C_3_H_10_T_1/2,_ was transfected with a TCF reporter of Wnt signalling activity (TOPFlash). C_3_H_10_T_1/2_ cells were seeded at a density of 3.1 × 10^4^ cells/cm^2^ and co-transfected with 0.03 μg M50 (reporter) or M51 (negative control) Super 8xTOPFlash and 0.003 μg phRL-CMV (Renilla reporter, transfection control) using Lipofectamine/Plus Reagent (Invitrogen). After 24 h, cells were treated with 20 mM LiCl, 20 mM NaCl, 200 ng/ml Wnt3a (Biotechne) or 10 nM EGF recombinant proteins for 24 h. Luciferase activity was measured using a Dual Luciferase Reporter Assay Kit (Promega) using a Dynex illuminometer.

### 2.9 Design of experiments (DoE)-based analysis of combined EGF/Wnt effects

The DoE was designed using Minitab software, using low, medium and high values of 0, 50 and 100 nM for EGF and 0, 300 and 600 ng/ml for Wnt3a. Y201 hTERT-MSC-EGFP reporter cells were plated in a 96-well plate (3.1 × 10^4^ cells/ cm^2^) and left to adhere for 24 h. The medium was then replaced with growth factor containing medium in a randomised order. After 48 h, cells were lysed in 0.1% Triton X-100 and read in a fluorimeter (excitation 485 nm; emission 520 nm). Data were analysed using Minitab using the response surface method.

### 2.10 Edu assay of cell proliferation

Primary MSCs were seeded at a density of 1 × 10^4^ cells/cm^2^ onto glass coverslips and allowed to adhere overnight in MSC basal medium. Following this, cells were incubated overnight in synchronisation medium. Cells were then exposed to 10 nM EGF alone or in combination with 10 μM IWR-1, or 200 ng/ml Wnt3a plus 10 μM EdU substrate. After 48 h EdU detection was performed according to the manufacturer's instructions (Click-iT® EdU Imaging Kit, Invitrogen). Cells were counterstained with DAPI before mounting with Prolong Gold Antifade Mountant. Samples were visualised using the LSM710 confocal microscopy system and images were quantified using Image J.

### 2.11 RNA-Seq analysis

200,000 primary human MSCs at p4 were plated into two wells of three 6 well plates in triplicate. Cells were allowed to adhere overnight in MSC basal medium before stimulation for 24 h with MSC basal medium (control), 10 nM EGF alone, or in combination with 10 μM IWR-1. Equivalent volumes of carrier, dH_2_O for EGF and DMSO for IWR-1, were added where relevant. After 24 h, total cellular RNA was extracted following the manufacturer's guidelines using a Nucleospin RNA II kit (Macherey-Nagel). Cells from two wells were pooled to increase yield. RNA quality was examined using an Agilent bioanalyser. 1 μg of good quality total RNA was used for mRNA sequencing library preparation using the NEBNext RNA Ultra Directional Library preparation kit for Illumina in conjunction with the NEBNext Poly(A) mRNA Magnetic Isolation Module (New England BioLabs Inc.), and NEBNext single 6 bp indexing primers, according to the manufacturer's instructions. Libraries were pooled at equimolar ratios, and the pool was sent for 2 × 150 base paired end sequencing on a HiSeq 3000 at the University of Leeds Next Generation Sequencing Facility.

### 2.12 Bioinformatics and identification of EGF gene targets

Sequence reads were trimmed to remove any adapter sequences using Cutadapt version 1.8.3 [34]. Trimmed reads were aligned to version GRCh38 of the human genome using HISAT2 [35]. Transcriptomes were assembled and gene expression quantified using the Tuxedo pipeline (version 2.2.1) [36]. Transcriptome assembly, merging, quantification and normalisation to fragments per kilobase per million mapped reads (FPKM) were performed using Cufflinks, Cuffmerge, Cuffquant and Cuffnorm, respectively. An analysis of variance model with experimental condition (Control, EGF, EGF + IWR-1) as factor was fit to the FPKM values in the R stats package [37], followed by Tukey HSD test to calculate adjusted *p*-values to estimate which groups differed. Target genes for gene set enrichment analysis (GSEA) were identified as either having *p* < .05 in [Control versus EGF] and [Control versus EGF + IWR-1], but not [EGF versus EGF + IWR-1] (classical gene targets, gene set 1), or by having p < .05 in [Control versus EGF] and [EGF versus EGF + IWR-1], but not [Control versus EGF + I] (EGF/β-catenin gene targets, gene set 2). GSEA was performed using the Molecular Signature Database (MSigDB) web tool (http://software.broadinstitute.org/gsea) [38] and run against the “Hallmarks” and “Gene Ontology: Biological Processes” to produce lists of gene sets associated with those genes.

### 2.13 Statistical analysis

Details of the statistical analyses performed throughout this work can be found in the Supplemental Material.

## 3 Results

### 3.1 RTK, ILK and β-catenin signalling are interrelated in human MSCs

Interest in EGF/β-catenin signalling cross-talk was first stimulated by global gene expression analyses of four immortalised human clonal MSC lines (hTERT-MSCs; identified as Y101, Y201, Y102 and Y202) that showed distinct functional differences [6]. Several significantly differentially expressed pathways across the four MSC lines, including EGF/RTK, integrin, Wnt and ILK signalling were identified [6]. Pathway analysis of the gene expression profiles revealed a highly enriched “focal adhesion” network with many differentially expressed genes in cell-matrix and cytokine/growth factor-receptor interactions, several connections via ILK, and increased transcript expression of EGF, PDGFα and ILK in the Y102 and Y202 MSC lines [6].

**Fig. S1:**
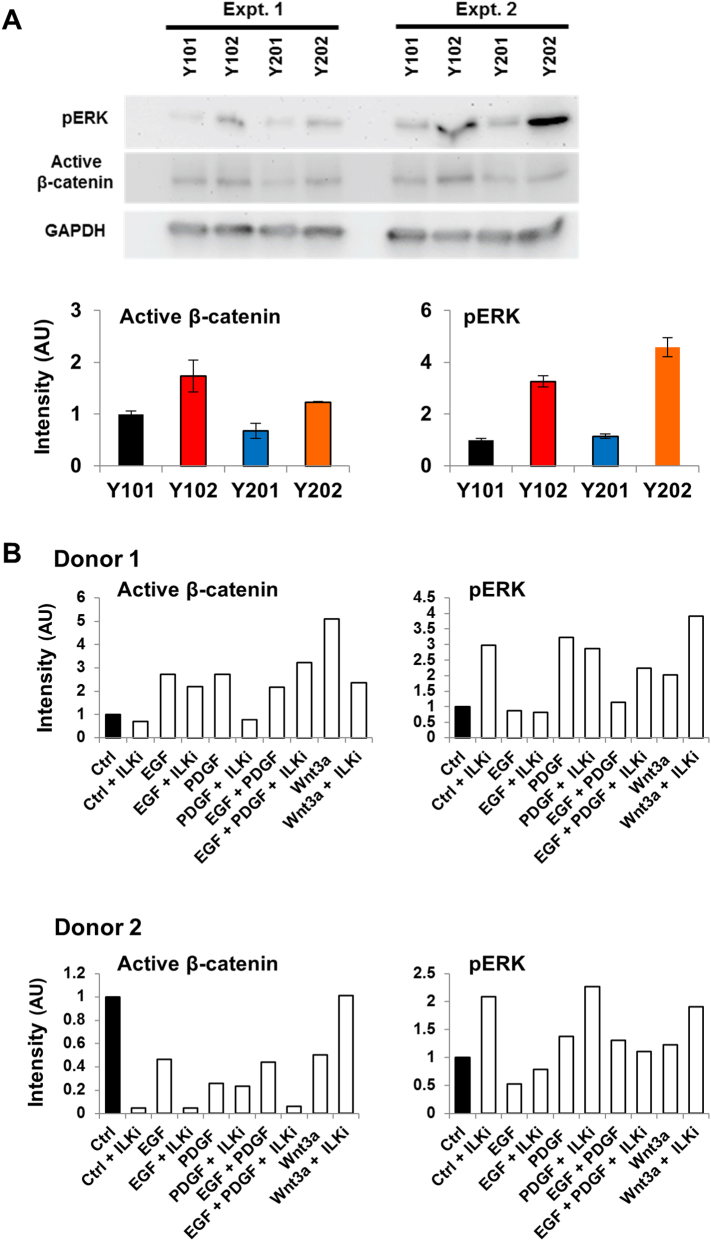
Basal RTK signalling levels vary in immortalised human MSCs and are influenced by ILK signalling. (A) Western blot analysis of basal expression of pERK and active β-catenin in different hTERT MSC clonal lines at two different passages (Expt. 1 and 2), densitometry is shown in the graphs, mean intensity ± SEM for two different passages (*n* = 2), normalised to GAPDH expression and made relative to expression in Y101 hTERT MSCs. (B) Densitometric analysis of band intensity normalised to GAPDH expression for Western Blots shown in Fig. 1D (Donors K102 and K96).

Protein was isolated from the hTERT-MSCs under basal conditions; the Y102 and Y202 lines expressed increased levels of activated (dephosphorylated) β-catenin and pERK at both passages by western blotting compared to Y101 and Y201 MSC lines (Fig. S1A). Y202 MSCs, which showed high endogenous pERK and β-catenin levels, were selected for further analysis by treating with increasing concentrations of specific inhibitors of EGF receptor (EGFR), PDGF receptor (PDGFR) and ILK, which resulted in a dose-dependent reduction in active β-catenin protein levels (Fig. 1B). To examine the effects of RTK signalling inhibition on cell growth, hTERT-MSCs were treated for 24 and 72 h with EGFR and PDGFR inhibitors, before viable cell numbers were measured using the MTT assay. After 72 h, all hTERT-MSC lines showed a significant reduction in cell number following treatment with a combination of both EGFR and PDGFR inhibitors, indicating reduced growth rate in response to RTK inhibition and providing evidence of autocrine RTK signalling. Furthermore, in all MSC lines, EGFR inhibition alone was sufficient to reduce significantly growth rate, whereas the inhibitory effects of PDGFR blockade were only significant in the presence of EGF (Fig. 1C). These initial findings provided circumstantial evidence that RTK (EGF predominantly) and ILK signalling were linked to both β-catenin activation and hTERT-MSC proliferation.

To test for a direct association, human primary MSCs were used to ensure our observations were not an anomaly associated with the hTERT-MSCs. Primary MSCs were treated with EGF and PDGF alone or in combination with an ILK inhibitor (ILKi) and pERK and active β-catenin expression was measured by western blotting after 24 h. This confirmed that EGF and PDGF increased active β-catenin in primary MSCs; however inter-donor variation was notable with varying basal levels of active β-catenin expression (examples shown in Fig. 1D with quantification in S1B, compare levels in control lane 1), in a manner similar to the variation observed across the hTERT-MSC lines. Inhibition of ILK did reduce EGF and PDGF-induced activation of β-catenin and notably, blockade of ILK increased basal ERK phosphorylation (e.g. Fig. 1D, compare band intensities in lanes 1 and 2, Control versus Control plus ILKi).

### 3.2 Active β-catenin levels are increased in primary MSCs in response to EGF and integrin signalling

**Fig. 2:**
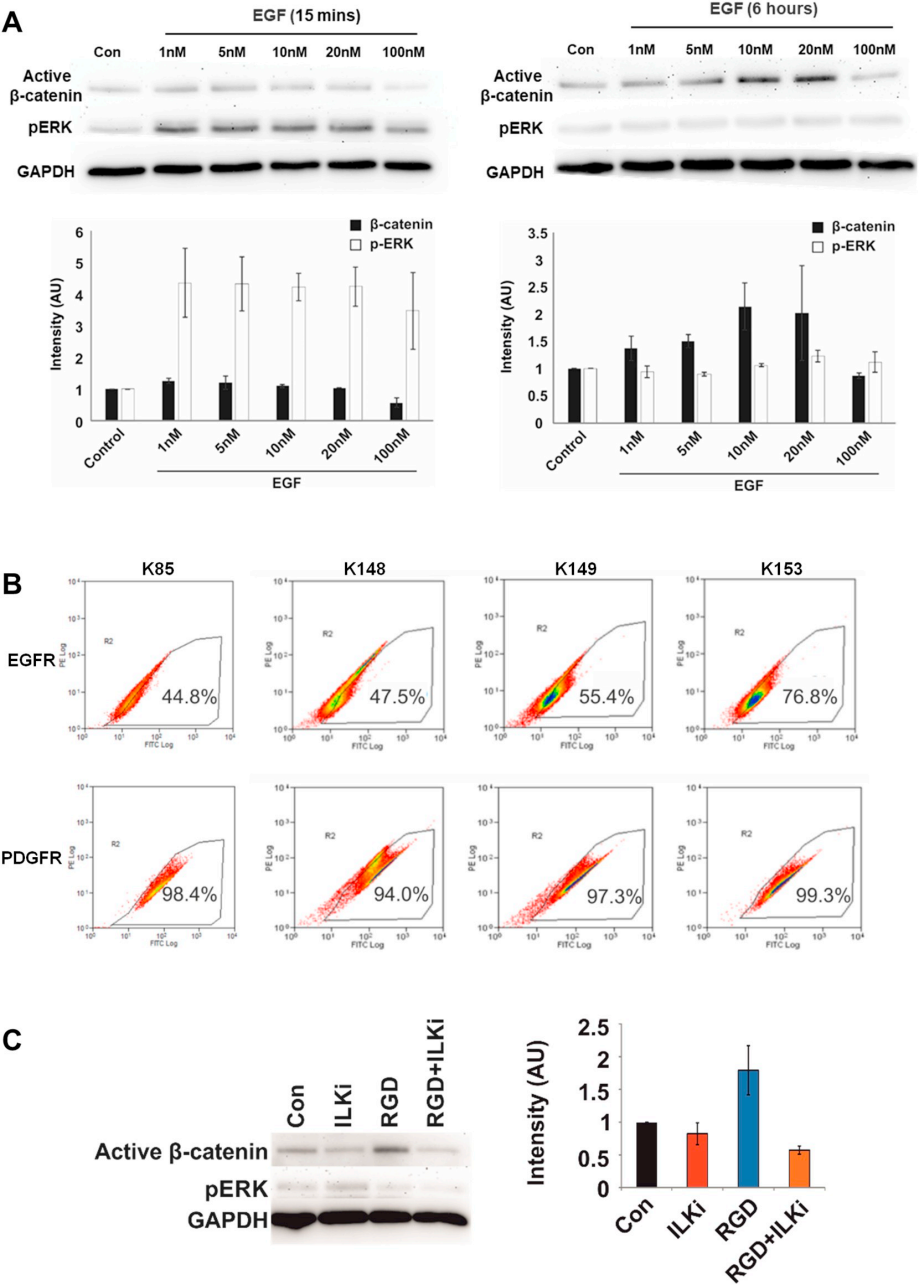
Active β-catenin levels are increased in primary MSCs in response to EGF and integrin signalling. (A) Western blot analysis of active β-catenin and pERK expression in primary MSCs in response to treatment with different concentrations of EGF for 15 min and 6 h. Below, densitometric analysis of band intensity normalised to GAPDH expression, mean intensity ± SEM for two different primary MSC donors (K135 and K139) (B) Flow cytometric analysis of basal EGFR and PDGFR expression in 4 primary MSC donors (K85, K148, K149 and K153). (C) Western blot analysis of active β-catenin and pERK expression in MSCs following treatment with an RGD peptide in the presence and absence of ILK inhibition (ILKi), example blot shown in left panel, graph in right panel shows densitometric analysis of β-catenin mean intensity ± SEM for two different primary MSC donors (K102 and K96). See also Fig. S2.

**Fig. S2:**
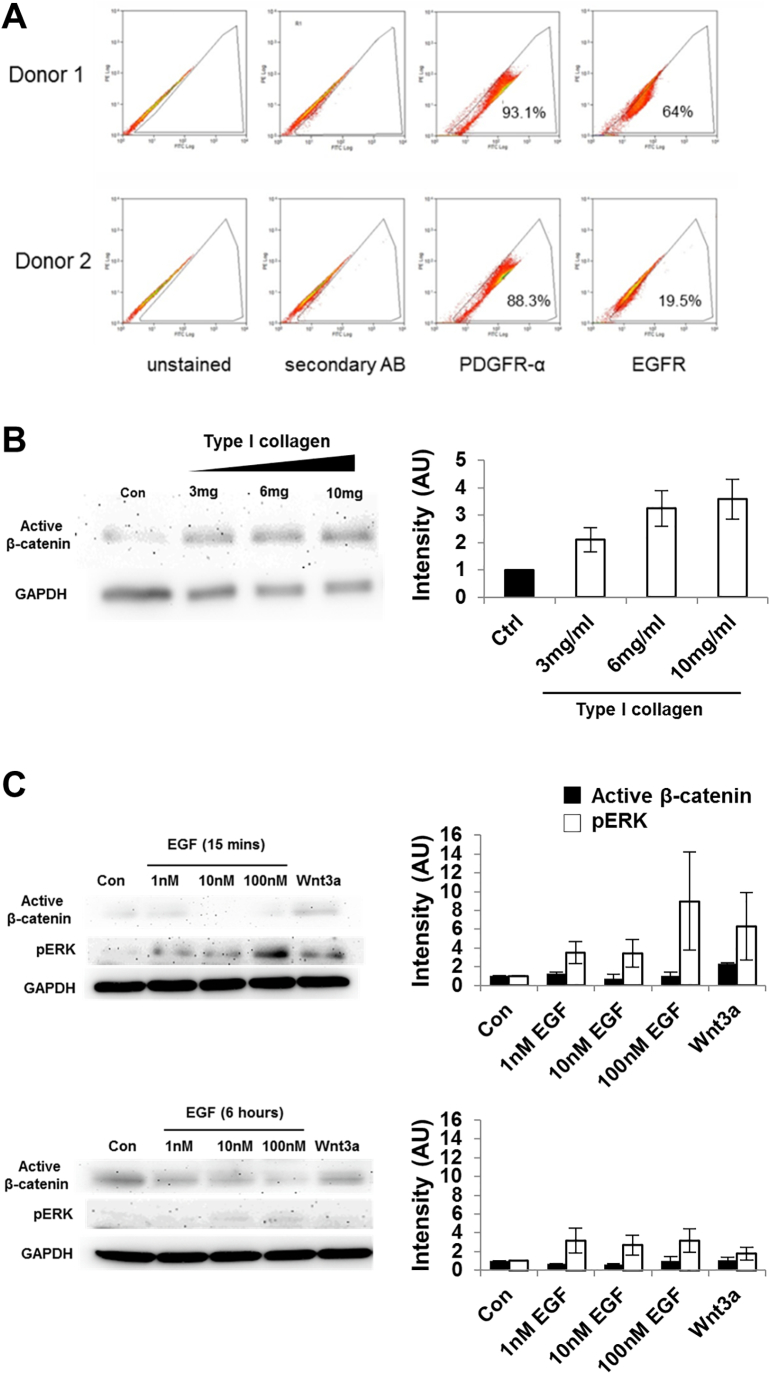
Active β-catenin expression in response to EGF and integrin signalling in human primary cells. (A) Flow cytometric analysis of basal EGFR and PDGFR expression in the 2 primary MSC donors (K102 and K96) examined by western blotting in Fig. 1D. (B) Western blot analysis of active β-catenin expression in primary MSCs seeded onto plates coated with type I collagen and cultured for 18 h. (Representative blot is shown on the left, and densitometric analysis is shown in the graph as mean band intensity ± SEM (n = 2, donors K102 and K96), normalised to expression of GAPDH). (C) Western blot analysis of active β-catenin expression in HDFs in response to treatment with EGF or Wnt3a as the positive control, a representative blot is shown on the left. Graphs show densitometric analysis of mean band intensity ± SEM (n = 2), normalised to expression of GAPDH.

We then focused on the time-course effects of EGF exposure on primary MSCs to delineate pERK/β-catenin pathway activation mechanisms. Protein was isolated from MSCs that had been treated with different concentrations of EGF for 15 min or 6 h and pERK and active β-catenin levels were measured by western blotting. An early transient increase in pERK expression was observed following 15 min exposure to all concentrations of EGF (1-100 nM), which returned to control levels by 6 h (Fig. 2A). Active β-catenin levels were unchanged after 15 min of EGF treatment, but an increase in active β-catenin was observed at 6 h (Fig. 2A). Flow cytometry was performed to determine expression of the EGFR and PDGFRα in four different human primary MSC donors; consistently high expression of PDGFRα was found. In contrast, the percentage of EGFR-expressing cells varied considerably, from 45 to 77% positivity in the four MSC samples examined (Fig. 2B). The MSCs analysed in Fig. 1D were recovered to determine EGFR/PDGFR expression by flow cytometry with variation again identified (Donor 1: 64% and 93%; Donor 2: 20% and 88% for EGFR and PDGFR respectively, Fig. S2A). Attempts were made to sort EGFR-positive MSCs but high EGFR levels were not maintained in culture and the populations reverted to variable EGFR expression (data not shown), which may be related to an autocrine EGF-EGFR negative feedback response and ligand-induced internalisation of the EGFR [39]. These findings suggest that EGF-induced β-catenin activation occurs later than ERK phosphorylation, it is concentration-dependent and that responses may be determined by the EGFR expression profile within a heterogeneous MSC population.

Considering the potential involvement of ILK in this signalling mechanism, cells were seeded onto plates coated with type I collagen, which is known to activate integrin signalling and drive ILK recruitment [40] and a dose-dependent increase in activation of β-catenin was observed (Fig. S2B). This effect was examined further by treating primary MSCs with an RGD peptide that mimics integrin binding. By western blotting RGD treatment increased levels of active β-catenin, which was blocked by ILK inhibition, thus supporting a role for the regulation of active β-catenin levels via ILK in primary human MSCs (Fig. 2B).

### 3.3 EGF-induced activation of β-catenin may be unique to MSCs

To determine if EGF-mediated activation of β-catenin occurred in other stromal cells, a related cell type, human dermal fibroblasts (HDFs), was treated with EGF. An increase in pERK expression following EGF exposure for 15 min was observed with expression returning to control levels by 6 h, consistent with MSC responses (Fig. S2C). Notably, active β-catenin protein levels remained similar to untreated controls at both time points, indicating that EGF-induced activation of β-catenin signalling does not occur in HDFs, and may be specific to MSCs (Fig. S2C).

### 3.4 EGF treatment drives active β-catenin nuclear translocation but does not induce TCF-mediated gene activation

**Fig. 3:**
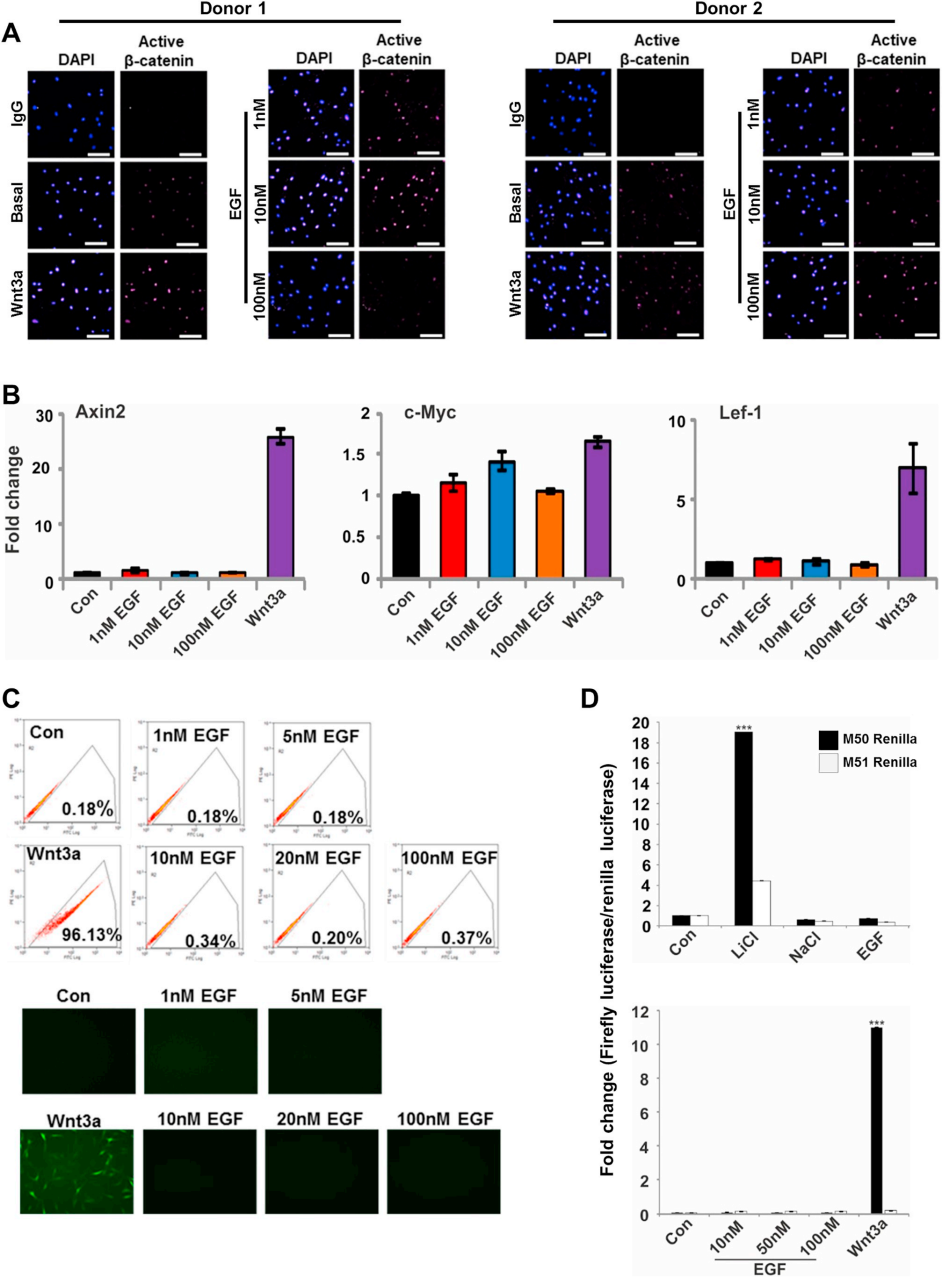
EGF treatment drives active β-catenin nuclear translocation but does not induce TCF-mediated gene activation. (A) Analysis of active β-catenin nuclear localisation following EGF (1-100 nM) treatment in two primary MSC donors (K135 and K139) by immunofluorescence detection (red = active β-catenin; blue = DAPI nuclear stain; IgG = antibody control; Basal = untreated control; Wnt3a = positive control). (B) QPCR analysis of β-catenin responsive genes Axin2, c-Myc and LEF-1 in primary MSCs after EGF or Wnt3a treatment. Values shown are mean expression ± SEM (*n* = 2, K135 and K139), normalised to GAPDH expression and relative to expression in untreated control samples. (C) Flow cytometric (upper panel) and immunofluorescent (lower panel) analysis of hTERT MSCs stably expressing a TCF-EGFP reporter following treatment with Wnt3a or EGF (1-100 nM). (D) TOPflash Wnt reporter (M50) assay of mouse mesenchymal cells (C_3_H_10_T_1/2_ cells) treated with the Wnt signalling activator, (LiCl with NaCl negative control, upper panel) or with Wnt3a (lower panel) and EGF values shown are mean fold change ± SEM (n = 6), normalised to control samples, *** = p < .001. M51 = negative control.

Following Wnt stimulation, β-catenin translocates to the nucleus to induce transcription of target genes by interacting with TCF/LEF transcription factors. Active β-catenin localisation in primary MSCs was examined following 6 h of EGF treatment. Immunocytochemistry revealed nuclear staining following treatment with the positive control Wnt3a, and increased nuclear β-catenin staining was also observed following treatment with EGF, though to varying degrees in different MSC samples (Fig. 3A, two MSC donors shown). QPCR was used to determine the effects of EGF treatment on known β-catenin regulated genes; Axin2, c-Myc and Lef-1. Whilst Wnt3a stimulation increased expression of Axin2 and Lef-1, but not c-Myc, EGF had no effect on the expression of the three genes at any concentration tested (Fig. 3B).

To investigate further, hTERT-MSCs (Y201) stably expressing a TCF-enhanced green fluorescent protein (EGFP) reporter [33] were used. Following treatment with Wnt3a, flow cytometric analysis revealed a significant increase in EGFP expression (96% positivity), indicating TCF activation. In contrast, negligible changes in EGFP expression (<0.5% positivity) were observed above controls at any EGF concentration (Fig. 3C, upper). These results were supported by fluorescent microscopic examination (Fig. 3C, lower). Finally, the mouse mesenchymal cell line, C_3_H_10_T_1/2_ was transiently transfected with a TCF/LEF-luciferase reporter and treated for 24 h with EGF, LiCl (a GSK3β inhibitor), NaCl (negative control) or Wnt3a. There was a significant increase in luciferase activity in cells treated with LiCl and Wnt3a compared to controls, whereas EGF treatment had no effect (Fig. 3D). Taken together these results indicate that EGF induces nuclear translocation of β-catenin, but does not drive TCF-mediated gene expression.

### 3.5 Activation of β-catenin by EGF does not influence Wnt-driven TCF-dependent gene expression

**Fig. 4:**
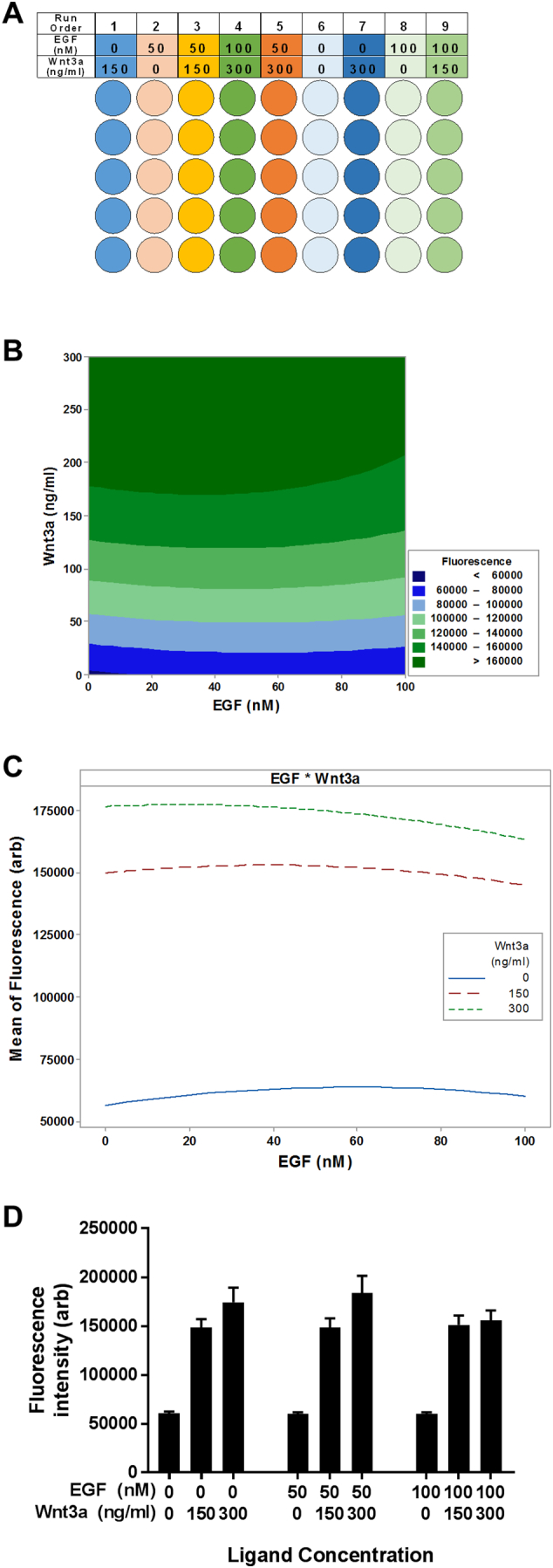
Activation of β-catenin by EGF is independent of Wnt signalling and does not drive TCF reporter expression. (A) Colour-coded schematic showing the 9-run experimental set-up with different combinations and concentration of EGF (0–100 nM) and Wnt3A (0–300 ng/ml). (B) Contour plot showing the effects of Wnt3a and EGF on the TCF-EGFP response. Dark green areas represent higher response. (C) Fitted predictive model of TCF reporter fluorescent response. Similar trends indicate insignificant effect of EGF on Wnt activation. (D) Bar graph in standard order of factorial design showing combined effects of Wnt and EGF in TCF reporter activity (fluorescence). See also Fig. S3.

**Fig. S3:**
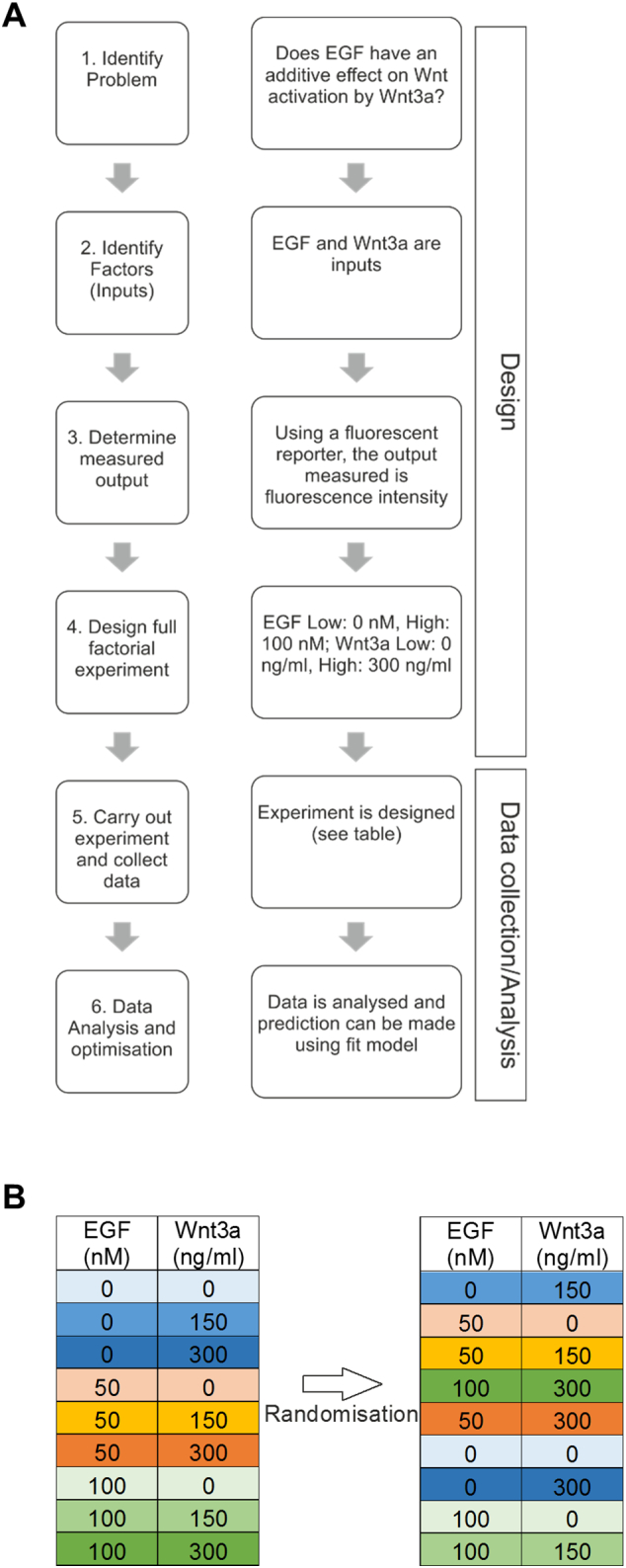
Diagram outlining the overall DoE process. (A) A DoE is designed like most other experiments by first identifying the question to be answered, then the factors to be tested (inputs) are determined (EGF and Wnt3a). The output measured is the response to a change in the inputs (TCF-EGFP reporter activity) and the high and low settings (levels) of the inputs are set. More levels can be included in DoEs, but for screening purposes, 2–3 is sufficient. Once set, a full factorial experiment is designed (see S3B). Data collection and analysis follows, where the experiment is carried out and trends are plotted using linear models. Initially, few midpoints are included in the experimental design in order to keep the number of experiments low. (B) Factor combinations are randomised in a DoE, helping to minimise effects of unaccounted for variables, such as systemic error in seeding density. One set of 9 runs is referred to as a block, where each block represents one replicate.

Until now, the effects of EGF and Wnt on β-catenin activation have been studied independently, although the possibility exists, and is perhaps more likely, that MSCs in vivo are exposed to different concentrations of these factors simultaneously, working in concert. For example, EGF may be able to augment Wnt-induced transcriptional events via cooperative β-catenin activation. To test this hypothesis, an efficient multi-factorial Design of Experiments (DoE) strategy was employed, where the hTERT-MSC TCF-EGFP reporter cells were exposed to different combinations and concentrations of EGF and Wnt3a in 9 parallel runs and TCF-mediated transcription (EGFP fluorescence) was determined by fluorimetry (Fig. 4A, S3A and S3B). These experiments conclusively demonstrated that EGF does not affect Wnt-induced transactivation. Contour plots of the effect of Wnt3a and EGF on EGFP fluorescence indicated that regardless of EGF concentration, Wnt activation occurred in a dose-dependent manner (Fig. 4B). The fitted means of fluorescent response indicated similar trends, providing strong evidence that there was no interaction between Wnt3a and EGF (*p* = .382). The effect of EGF alone on the response was insignificant (*p* = .486), therefore differences between EGF concentrations were within error (Fig. 4C and D). Collectively, these findings demonstrate that activation and nuclear translocation of β-catenin by EGF is independent of the Wnt-β-catenin-TCF pathway.

### 3.6 EGF controls MSC proliferation via ILK and β-catenin activation

**Fig. 5:**
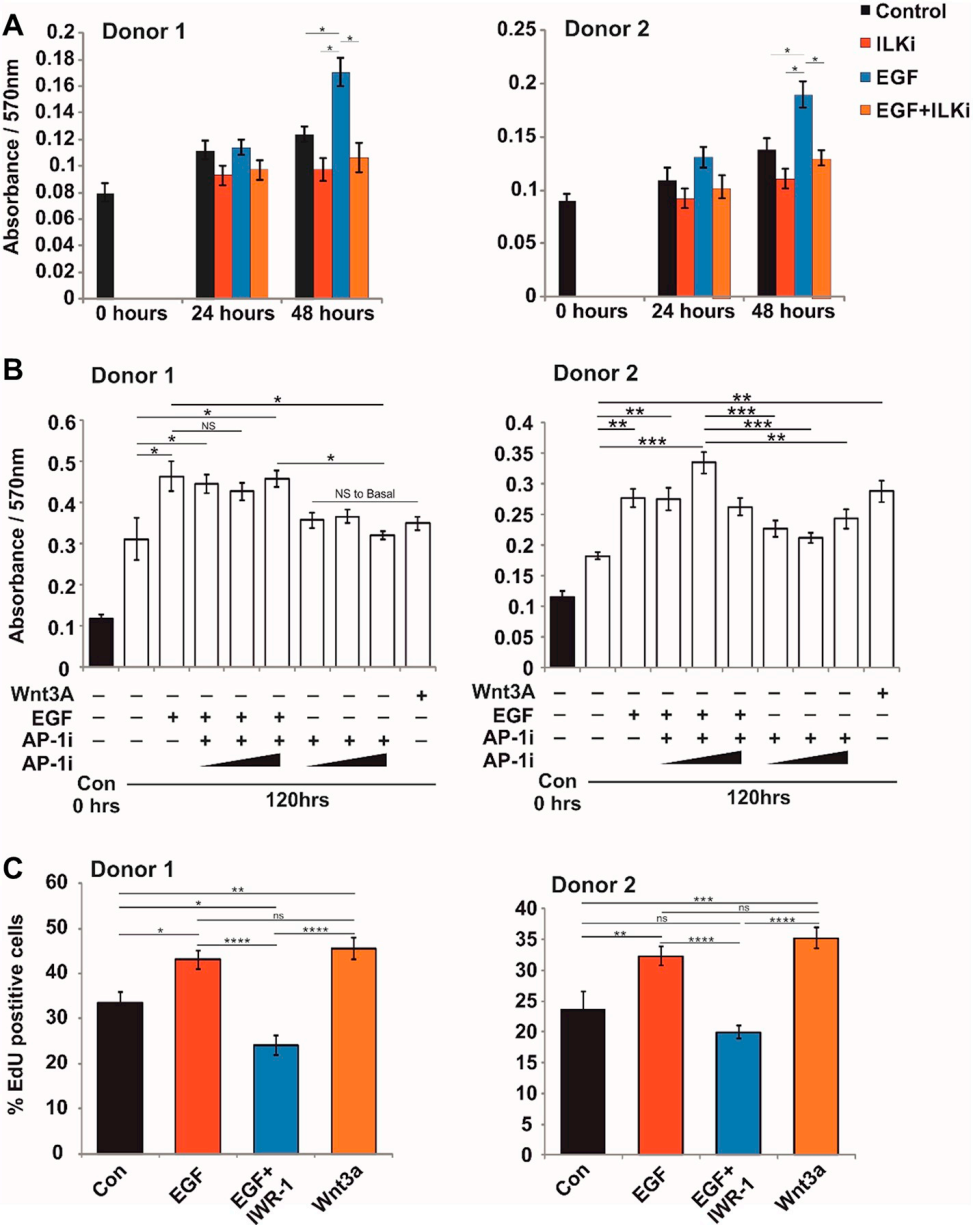
EGF controls MSC proliferation via ILK and β-catenin activation. (A) MTT assay of viable cell numbers in two primary MSC donors following 24 and 48 h treatment with EGF and ILKi, graphs show mean absorbance ± SEM (n = 6), * = *p* < .05. (B) MTT assay of viable cell numbers in two primary MSC donors following 120 h treatment with EGF and increasing concentrations of an inhibitor of the AP-1 complex (AP-1i, concentrations = 17 nM, 34 nM and 68 nM), graphs show mean absorbance ± SEM (n = 6), * = p < .05, ** < 0.01 *** < 0.001. (C) EdU proliferation assay in two primary MSC donors following 48 h treatment with EGF, IWR-1 and Wnt3A, graphs show mean percentage EdU positive cells ± SEM (*n* = 23), * = p < .05, ** < 0.01 *** < 0.001, **** < 0.0001. Donor 1 = K168, Donor 2 = K185. See also Fig. S4.

**Fig. S4:**
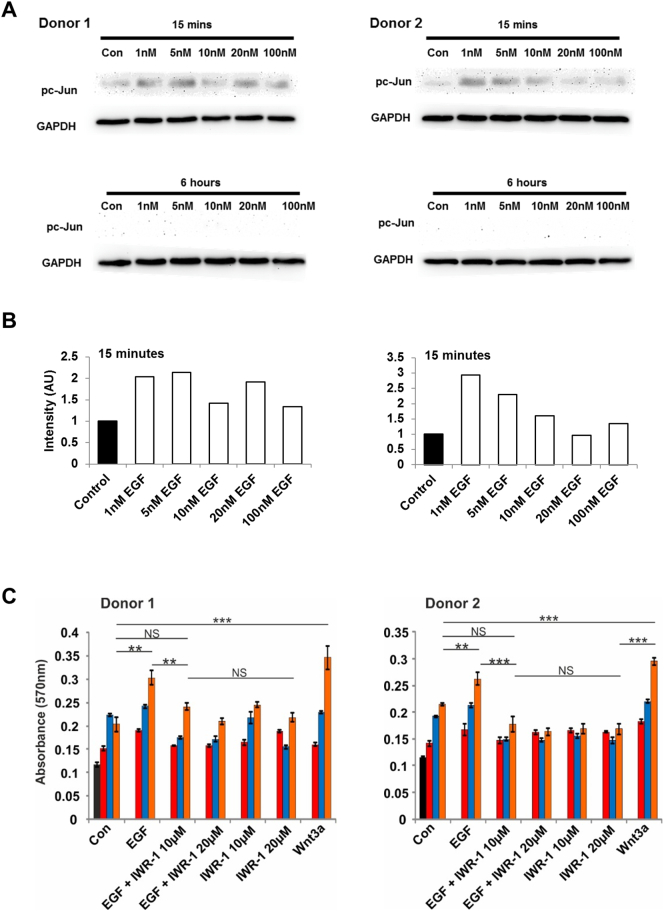
EGF treatment drives transient early expression of phospho c-Jun and proliferation in primary MSCs, which is reduced by blockade β-catenin nuclear translocation with IWR-1. (A) Western blot analysis of phospho c-Jun (pc-Jun) expression in 2 primary MSC donors (K135 and K139) treated with EGF for 15 min or 6 h. (B) Densitometric analysis of Westerns blots shown above (15 min), graphs show band intensity normalised to expression of GAPDH. (C) MTT assay of viable cell numbers in two primary MSC donors (K168 and K185) following 48–120 h treatment with EGF and increasing concentrations of IWR-1, graphs show mean absorbance ± SEM (*n* = 6), ** < 0.01 *** < 0.001.

Given the pronounced effects of EGFR inhibition on hTERT-MSC numbers, the role of the EGF-ILK-β-catenin pathway on proliferation in primary MSCs was analysed: viable MSC numbers were increased following 48 h of EGF treatment, but this effect was negated by ILK inhibition (Fig. 5A). AP-1 is a downstream target of EGF and pERK signalling and a known activator of cell proliferation. We demonstrated by western blot analysis that EGF induced early and transient expression of pc-Jun, which is part of the AP-1 complex (Fig. S4A and S4B). MSCs were treated with increasing concentrations of an AP-1 inhibitor in the presence and absence of EGF and again found that EGF significantly increased viable cell numbers of MSCs, and that this stimulatory effect was not affected by AP-1 inhibition (Fig. 5B) indicating that EGF-induced proliferation of MSCs was not mediated by the AP-1 complex.

The role of the β-catenin pathway in EGF-induced proliferation of primary MSCs was probed using IWR-1, a compound known to stabilise the APC/Axin/GSK-3β destruction complex through interaction with Axin2, thereby preventing β-catenin nuclear translocation. Primary MSCs were treated for 48 h with EGF alone or in combination with IWR-1, and proliferation measured by EdU incorporation and cell numbers by MTT assay. IWR-1 significantly reduced the proliferation of MSCs treated with EGF at 48 h (Fig. 5C) and numbers of viable cells (Fig. S4C), demonstrating that β-catenin activation is required for EGF-induced proliferation of primary human MSCs.

### 3.7 Classical EGF and EGF/β-catenin signalling pathways regulate the expression of distinct gene sets

**Fig. 6:**
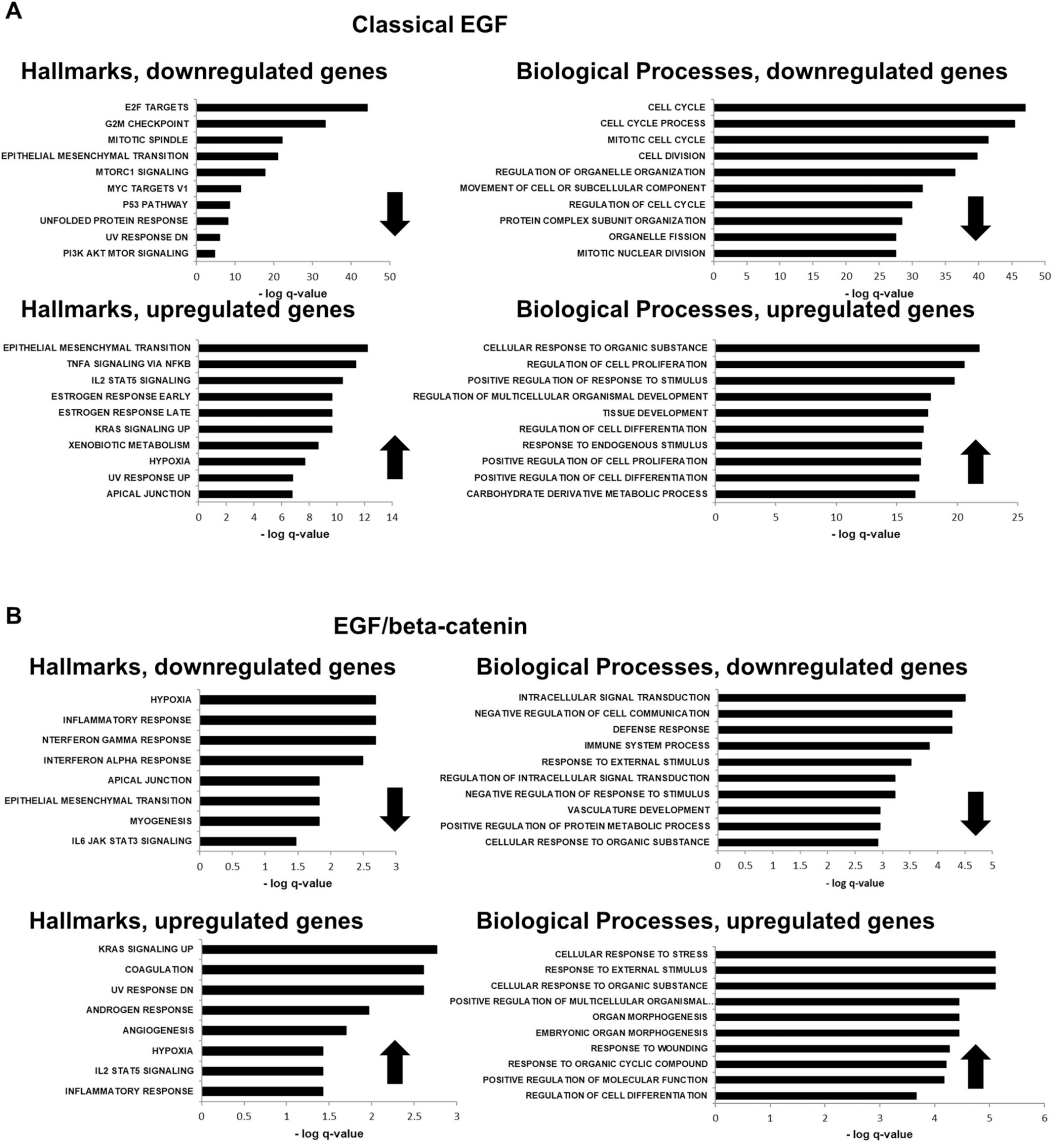
Classical EGF and EGF/β-catenin signalling pathways control the expression of distinct gene sets. (A) GSEA and GO Term bioinformatics analysis showing Hallmarks and Biological Processes significantly downregulated and upregulated in response to classical EGF signalling. (B) GSEA and GO Term bioinformatics analysis showing Hallmarks and Biological Processes significantly downregulated and upregulated in response to EGF/β-catenin signalling. See also Fig. S5 and Table S1.

**Fig. S5:**
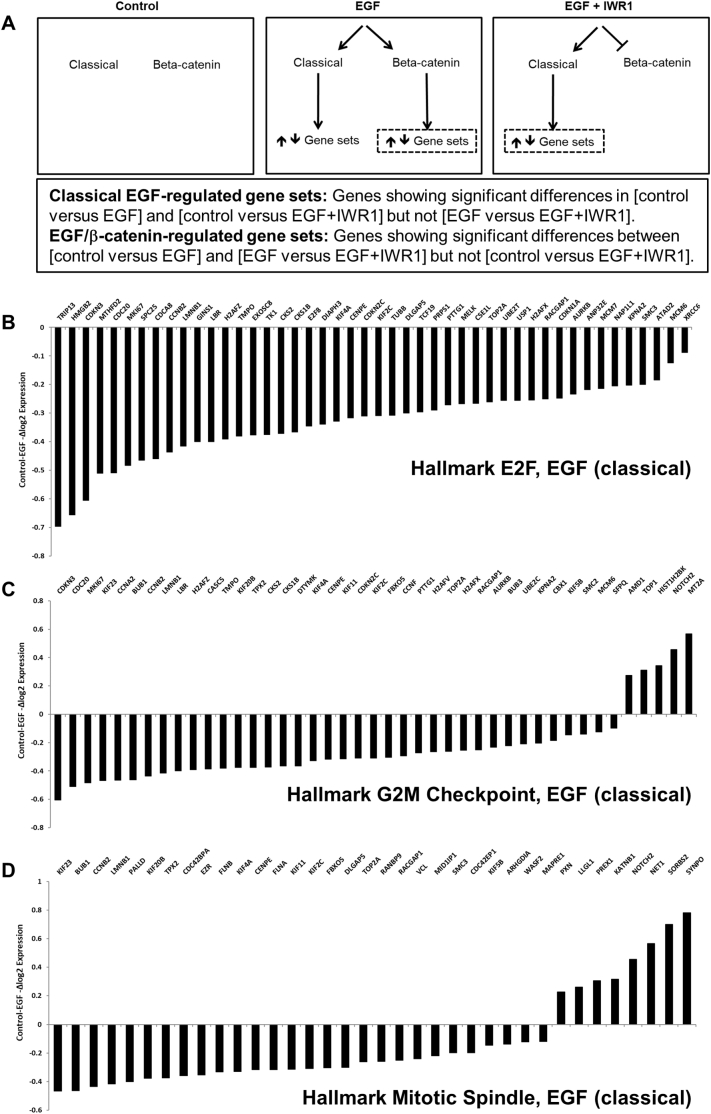
Identifying classical EGF and EGF/β-catenin signalling pathways in MSCs. (A) Experimental strategy to determine Classical EGF- and EGF/β-catenin-regulated gene sets. (B, C, D). Classical EGF signalling downregulates genes associated with the cell cycle in MSCs. Graphs show effects of classical EGF activation on individual genes associated with the most significantly downregulated Hallmarks; E2F Targets (B); G2M Checkpoint (C) and Mitotic Spindle (D).

An experimental strategy was devised to determine the different gene sets that were regulated by “classical” EGF signalling and the EGF/β-catenin signalling pathway (Fig. S5A). Primary human MSCs were treated (in triplicate) with EGF alone or EGF with IWR-1 for 24 h and changes in gene expression were determined by RNA-Seq analysis, compared to untreated controls. By examining statistical differences between the treatment groups, classical EGF gene targets, those showing significant (*P* < .05) differences in [control versus EGF] and [control versus EGF + IWR1] but not [EGF versus EGF + IWR1] were identifiable; likewise, EGF/β-catenin gene targets were identified as showing significant differences between [control versus EGF] and [EGF versus EGF + IWR1] but not [control versus EGF + IWR1]. These comparisons generated lists of 879 classical EGF-regulated genes and 155 EGF/β-catenin-regulated genes that were subjected to Gene Set Enrichment Analysis (GSEA: Hallmarks and Gene Ontology: Biological Processes). Different, significantly enriched Hallmarks and Biological Processes (BP) were identified for the classical EGF and EGF/β-catenin pathways (Table S1). Independent analysis of upregulated and downregulated genes showed that the most significantly downregulated classical EGF-regulated pathways were associated with cell proliferation including the Hallmarks E2F Targets; G2M Checkpoint, Mitotic Spindle and GO Terms Cell Cycle, Cell Cycle Process; Mitotic Cell Cycle; Cell Division (Fig. 6A and Fig. S5B-D). Upregulated classical EGF-regulated pathways were associated largely with cell defence responses, tissue development and differentiation (Fig. 6A). In contrast, Hallmarks and Biological Processes that were significantly influenced by the EGF/β-catenin pathway included immune responses, KRas signalling and stress responses, amongst others (Fig. 6B).

**Fig. 7:**
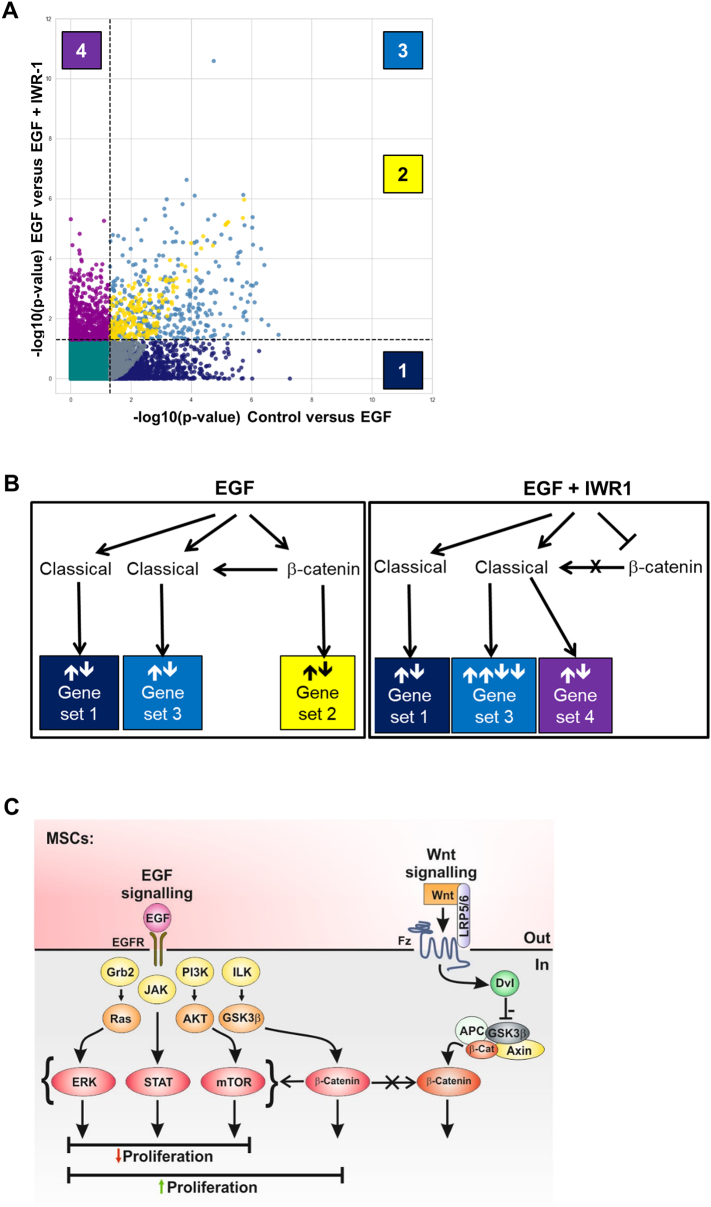
EGF regulates at least four distinct gene sets. (A) Scatterplot of differentially expressed genes following EGF exposure for 24 h, with or without IWR-1. EGF can signal via classical (1, dark blue) and EGF/β-catenin (2, yellow) pathways. The expression of other EGF targets can be up- or down-regulated (3, light blue) or abolished (4, purple) by activated β-catenin. (B) Schematic to summarise the different EGF-regulated gene sets. (C) Proposed model of EGF signalling pathways in MSCs.

A scatter plot was used to display the data based on differences across the Control/EGF/EGF + IWR-1 treatment groups (Fig. 7A). It should be noted that these separations were based on a significance threshold of *P* < .05, so some false positive and negatives may be included. Visualising the data in this manner clearly identified at least four differentially regulated sets of genes; the classical EGF-regulated genes are represented as dark blue spots (gene set 1, lower right quadrant, Fig. 7A) and the EGF/β-catenin-regulated genes as yellow spots (gene set 2, upper right quadrant, Fig. 7A). There were also gene sets whose expression appeared to be influenced by the EGF/β-catenin pathway; classical-regulated genes that were either further increased or decreased by an active EGF/β-catenin pathway (gene set 3, light blue spots, upper right quadrant, Fig. 7A) and classical EGF-regulated genes whose expression was dependent on an active EGF/β-catenin pathway (gene set 4, purple spots, upper left quadrant, Fig. 7A). We have proposed a model as a potential mechanism by which this regulatory control may occur (Fig. 7B) with an updated interpretation of EGF signalling pathways in MSCs (Fig. 7C).

## 4 Discussion

By attempting to understand the roles of growth factor and integrin signalling in human MSCs, we have identified a Wnt ligand-independent mechanism of β-catenin activation, mediated by EGF. β-catenin activation via EGF/EGFR induction has been demonstrated in a number of studies using cancer cells. For example, the internalisation of E-cadherin following EGF treatment results in the dissociation of β-catenin from the cadherin complex, nuclear translocation and activation of TCF/LEF gene targets including c-Myc and cyclin D1 [41,42]. This may also be aided by the co-binding of embryonic pyruvate kinase M2 (PKM2), which is present at high levels in multiple human cancer lines [20]. It is important to note that cancer cells frequently over-express EGFR with defective negative feedback responses [39]; characteristics that are not a feature of normal human cells. Related reports using human urothelial cells have also pointed to a relationship between EGF and Wnt signalling, albeit in this case operating together in a positive feedback mechanism to control proliferation and again via TCF activation [43]. The data presented here demonstrate a role for EGF signalling in β-catenin activation and nuclear translocation in normal human MSCs. However, this mechanism of signalling does not appear to be a feature of all stromal cells as EGF treatment of HDFs did not drive accumulation of active β-catenin. Our data implicate the involvement of ILK in EGF-induced active β-catenin accumulation in primary MSCs. This alternative route of β-catenin activation may be due to the interaction of RTK adaptor proteins on the intracellular face of the plasma membrane, leading to activation of the ILK kinase domain and subsequent inhibition of GSK-3β [19]. Unlike previous work in other cell types, we did not observe increased TCF-driven gene transcription following EGF-mediated β-catenin nuclear translocation in MSCs. Furthermore, we clearly demonstrated by DoE that EGF could not influence Wnt-induced TCF reporter activity. Indeed, maximum reporter levels were achieved with no/low EGF and high Wnt concentrations, providing strong evidence that the EGF/ β-catenin signalling pathway in MSCs does not act via TCF, but operates through distinct mechanism to regulate the expression of different target gene sets. Bioinformatics analysis of the RNA-Seq data identified at least four distinct gene sets that were exclusively regulated either by classical EGF or EGF/β-catenin pathways, as well genes that were co-regulated by both pathways, demonstrating the complexity of this signal transduction mechanism. These analyses also highlighted a potential mechanism through which EGF controls MSC proliferation. Blockade of β-catenin activation prevented EGF-induced proliferation by enhancing the inhibitory effects of classical EGF signalling on cell cycle-related pathways.

We observed inter-donor variation as well as intra-donor variation (using the hTERT-MSC clonal lines), which may be linked to varying basal levels of endogenous EGF and/or β-catenin signalling activity across MSC populations and reflect authentic biological function. Indeed this biological variation may have obscured the discovery of this signalling pathway in MSCs. We have used primary MSCs from 10 donors, plus four MSC clonal lines, throughout the course of this work in an attempt to aid accurate interpretation of the data and primary MSCs were used at similar passages (up to a maximum of passage 5) to help minimise variations due to in vitro culture conditions. The importance of these findings extends beyond a fundamental understanding of MSC biology. Many previous studies, using both in vitro and in vivo methods have assessed β-catenin signalling in MSCs and highlighted its importance in bone formation. Perturbations in β-catenin signalling have resulted in greatly reduced bone formation [44], loss of osteogenic tissues and altered skeletal development [45], demonstrating a critical role for β-catenin in these processes. Along with numerous other studies [14,15,46,47], this previously published work considered the regulation of β-catenin activity in the context of canonical Wnt signalling and there is widespread interest in using modulators of Wnt/β-catenin to treat bone disease [48]. The findings presented here clearly demonstrate an alternative, Wnt-independent mechanism for β-catenin activation in human MSCs. There are reports that β-catenin can induce gene expression in a TCF/LEF-independent manner, through *E*-box element binding [49] and via vitamin D response elements [50,51], and similar mechanisms may be acting in MSC cultures. This study highlights the need for more detailed analysis of both classical and alternative routes for β-catenin activation to ensure that the integrated signalling mechanisms controlling MSC function are fully uncovered. These endeavours will also impact the development of new therapies for skeletal disorders, particularly those related to the use of Wnt signalling mimetics.

The following are the supplementary data related to this article.

Table S1 Classical EGF and EGF/β-catenin signalling pathways significantly enrich different gene sets in MSCs. Lists of most significantly differentially enriched Hallmarks for classical EGF and EGF/β-catenin signalling pathways (upper panels). Lists of most significantly differentially enriched Biological Processes for classical EGF and EGF/β-catenin signalling pathways (lower panels). Table S1

Supplementary material Image 1

## References

[1] Carstairs A., Genever P. (2014). Stem cell treatment for musculoskeletal disease. Curr. Opin. Pharmacol..

[2] Bluguermann C., Wu L., Petrigliano F., McAllister D., Miriuka S., Evseenko D.A. (2013). Novel aspects of parenchymal–mesenchymal interactions: from cell types to molecules and beyond. Cell Biochem. Funct..

[3] Pittenger M.F., MacKay A.M., Beck S.C., Jaiswal R.K., Douglas R., Mosca J.D., Moorman M.A., Simonetti D.W., Craig S., Marshak D.R. (1999). Multilineage potential of adult human mesenchymal stem cells. Science.

[4] Jones E., McGonagle D. (2008). Human bone marrow mesenchymal stem cells in vivo. Rheumatology (Oxford).

[5] Pevsner-Fischer M., Levin S., Zipori D. (2011). The origins of mesenchymal stromal cell heterogeneity. Stem Cell Rev..

[6] James S., Fox J., Afsari F., Lee J., Clough S., Knight C., Ashmore J., Ashton P., Preham O., Hoogduijn M., Ponzoni Rde A., Hancock Y., Coles M., Genever P. (2015). Multiparameter analysis of human bone marrow stromal cells identifies distinct immunomodulatory and differentiation-competent subtypes. Stem Cell Rep..

[7] Rubinfeld B., Albert I., Porfiri E., Fiol C., Munemitsu S., Polakis P. (1996). Binding of GSK3β to the APC-β-catenin complex and regulation of complex assembly. Science.

[8] Brunner E., Peter O., Schweizer L., Basler K. (1997). Pangolin encodes a Lef-1 homologue that acts downstream of Armadillo to transduce the Wingless signal in Drosophila. Nature.

[9] van de Wetering M., Cavallo R., Dooijes D., van Beest M., van Es J., Loureiro J., Ypma A., Hursh D., Jones T., Bejsovec A., Peifer M., Mortin M., Clevers H. (1997). Armadillo coactivates transcription driven by the product of the Drosophila segment polarity gene dTCF. Cell.

[10] Gong Y., Slee R.B., Fukai N., Rawadi G., Roman-Roman S., Reginato A.M., Wang H., Cundy T., Glorieux F.H., Lev D., Zacharin M., Oexle K., Marcelino J., Suwairi W., Heeger S., Sabatakos G., Apte S., Adkins W.N., Allgrove J., Arslan-Kirchner M., Batch J.A., Beighton P., Black G.C., Boles R.G., Boon L.M., Borrone C., Brunner H.G., Carle G.F., Dallapiccola B., De Paepe A., Floege B., Halfhide M.L., Hall B., Hennekam R.C., Hirose T., Jans A., Juppner H., Kim C.A., Keppler-Noreuil K., Kohlschuetter A., Lacombe D., Lambert M., Lemyre E., Letteboer T., Peltonen L., Ramesar R.S., Romanengo M., Somer H., Steichen-Gersdorf E., Steinmann B., Sullivan B., Superti-Furga A., Swoboda W., van den Boogaard M.J., Van Hul W., Vikkula M., Votruba M., Zabel B., Garcia T., Baron R., Olsen B.R., Warman M.L., G. Osteoporosis-Pseudoglioma Syndrome Collaborative (2001). LDL receptor-related protein 5 (LRP5) affects bone accrual and eye development. Cell.

[11] Boland G.M., Perkins G., Hall D.J., Tuan R.S. (2004). Wnt 3a promotes proliferation and suppresses osteogenic differentiation of adult human mesenchymal stem cells. J. Cell. Biochem..

[12] De Boer J., Wang H.J., Van Blitterswijk C. (2004). Effects of Wnt signaling on proliferation and differentiation of human mesenchymal stem cells. Tissue Eng..

[13] Etheridge S.L., Spencer G.J., Heath D.J., Genever P.G. (2004). Expression profiling and functional analysis of wnt signaling mechanisms in mesenchymal stem cells. Stem Cells.

[14] Cook D.A., Fellgett S.W., Pownall M.E., O'Shea P.J., Genever P.G. (2014). Wnt-dependent osteogenic commitment of bone marrow stromal cells using a novel GSK3beta inhibitor. Stem Cell Res..

[15] Bain G., Muller T., Wang X., Papkoff J. (2003). Activated beta-catenin induces osteoblast differentiation of C3H10T1/2 cells and participates in BMP2 mediated signal transduction. Biochem. Biophys. Res. Commun..

[16] Rawadi G., Vayssiere B., Dunn F., Baron R., Roman-Roman S. (2003). BMP-2 controls alkaline phosphatase expression and osteoblast mineralization by a Wnt autocrine loop. J. Bone Miner. Res..

[17] Dedhar S., Williams B., Hannigan G. (1999). Integrin-linked kinase (ILK): a regulator of integrin and growth-factor signalling. Trends Cell Biol..

[18] Delcommenne M., Tan C., Gray V., Rue L., Woodgett J., Dedhar S. (1998). Phosphoinositide-3-OH kinase-dependent regulation of glycogen synthase kinase 3 and protein kinase B/AKT by the integrin-linked kinase. Proc. Natl. Acad. Sci. U. S. A..

[19] Naska S., Park K.J., Hannigan G.E., Dedhar S., Miller F.D., Kaplan D.R. (2006). An essential role for the integrin-linked kinase-glycogen synthase kinase-3 beta pathway during dendrite initiation and growth. J. Neurosci..

[20] Yang W., Xia Y., Ji H., Zheng Y., Liang J., Huang W., Gao X. (2011). Nuclear PKM2 regulates β-catenin transactivation upon EGFR activation. Nature.

[21] Krejci P., Aklian A., Kaucka M., Sevcikova E., Prochazkova J., Masek J.K., Mikolka P., Pospisilova T., Spoustova T., Weis M., Paznekas W.A., Wolf J.H., Gutkind J.S., Wilcox W.R., Kozubik A., Jabs E.W., Bryja V., Salazar L., Vesela I., Balek L. (2012). Receptor tyrosine kinases activate canonical WNT/beta-catenin signaling via MAP kinase/LRP6 pathway and direct beta-catenin phosphorylation. PLoS One.

[22] Zhang X., Zhu J., Li Y., Lin T., Siclari V.A., Chandra A., Candela E.M., Koyama E., Enomoto-Iwamoto M., Qin L. (2013). Epidermal growth factor receptor (EGFR) signaling regulates epiphyseal cartilage development through beta-catenin-dependent and -independent pathways. J. Biol. Chem..

[23] Greenblatt M.B., Shin D.Y., Oh H., Lee K.-Y., Zhai B., Gygi S.P., Lotinun S., Baron R., Liu D., Su B., Glimcher L.H., Shim J.-H. (2016). MEKK2 mediates an alternative β-catenin pathway that promotes bone formation. Proc. Natl. Acad. Sci..

[24] Sibilia M., Wagner B., Hoebertz A., Elliott C., Marino S., Jochum W., Wagner E.F. (2003). Mice humanised for the EGF receptor display hypomorphic phenotypes in skin, bone and heart. Development.

[25] Fisher M.C., Clinton G.M., Maihle N.J., Dealy C.N. (2007). Requirement for ErbB2/ErbB signaling in developing cartilage and bone. Develop. Growth Differ..

[26] Zhang X., Siclari V.A., Lan S., Zhu J., Koyama E., Dupuis H.L., Enomoto-Iwamoto M., Beier F., Qin L. (2011). The critical role of the epidermal growth factor receptor in endochondral ossification. J. Bone Miner. Res..

[27] Zhang X., Tamasi J., Lu X., Zhu J., Chen H., Tian X., Lee T.C., Threadgill D.W., Kream B.E., Kang Y., Partridge N.C., Qin L. (2011). Epidermal growth factor receptor plays an anabolic role in bone metabolism in vivo. J. Bone Miner. Res..

[28] Krampera M., Pasini A., Rigo A., Scupoli M.T., Tecchio C., Malpeli G., Scarpa A., Dazzi F., Pizzolo G., Vinante F. (2005). HB-EGF/HER-1 signaling in bone marrow mesenchymal stem cells: inducing cell expansion and reversibly preventing multilineage differentiation. Blood.

[29] Tamama K., Fan V.H., Griffith L.G., Blair H.C., Wells A. (2006). Epidermal growth factor as a candidate for ex vivo expansion of bone marrow-derived mesenchymal stem cells. Stem Cells.

[30] Yu S., Geng Q., Ma J., Sun F., Yu Y., Pan Q., Hong A. (2013). Heparin-binding EGF-like growth factor and miR-1192 exert opposite effect on Runx2-induced osteogenic differentiation. Cell Death Dis..

[31] Liu X., Qin J., Luo Q., Bi Y., Zhu G., Jiang W., Kim S.H., Li M., Su Y., Nan G., Cui J., Zhang W., Li R., Chen X., Kong Y., Zhang J., Wang J., Rogers M.R., Zhang H., Shui W., Zhao C., Wang N., Liang X., Wu N., He Y., Luu H.H., Haydon R.C., Shi L.L., Li T., He T.C., Li M. (2013). Cross-talk between EGF and BMP9 signalling pathways regulates the osteogenic differentiation of mesenchymal stem cells. J. Cell. Mol. Med..

[32] Boonanantanasarn K., Lee H.L., Baek K., Woo K.M., Ryoo H.M., Baek J.H., Kim G.S. (2015). EGF inhibits Wnt/β-catenin-induced osteoblast differentiation by promoting β-catenin degradation. J. Cell. Biochem..

[33] Saleh F., Carstairs A., Etheridge S.L., Genever P. (2016). Real-time analysis of endogenous Wnt signalling in 3D mesenchymal stromal cells. Stem Cells Int..

[34] Martin M. (2011). Cutadapt removes adapter sequences from high-throughput sequencing reads. EMBnet J..

[35] Kim D., Langmead B., Salzberg S.L. (2015). HISAT: a fast spliced aligner with low memory requirements. Nat. Methods.

[36] Trapnell C., Roberts A., Goff L., Pertea G., Kim D., Kelley D.R., Pimentel H., Salzberg S.L., Rinn J.L., Pachter L. (2012). Differential gene and transcript expression analysis of RNA-seq experiments with TopHat and Cufflinks. Nat. Protoc..

[37] R Core Team (2016). R: A Language and Environment for Statistical Computing.

[38] Subramanian A., Tamayo P., Mootha V.K., Mukherjee S., Ebert B.L., Gillette M.A., Paulovich A., Pomeroy S.L., Golub T.R., Lander E.S., Mesirov J.P. (2005). Gene set enrichment analysis: a knowledge-based approach for interpreting genome-wide expression profiles. Proc. Natl. Acad. Sci..

[39] Avraham R., Yarden Y. (2011). Feedback regulation of EGFR signalling: decision making by early and delayed loops. Nat. Rev. Mol. Cell Biol..

[40] Leyme A., Bourd-Boittin K., Bonnier D., Falconer A., Arlot-Bonnemains Y., Theret N. (2012). Identification of ILK as a new partner of the ADAM12 disintegrin and metalloprotease in cell adhesion and survival. Mol. Biol. Cell.

[41] Lu Z., Ghosh S., Wang Z., Hunter T. (2003). Downregulation of caveolin-1 function by EGF leads to the loss of E-cadherin, increased transcriptional activity of β-catenin, and enhanced tumor cell invasion. Cancer Cell.

[42] Lee C.H., Hung H.W., Hung P.H., Shieh Y.S. (2010). Epidermal growth factor receptor regulates beta-catenin location, stability, and transcriptional activity in oral cancer. Mol. Cancer.

[43] Georgopoulos N.T., Kirkwood L.A., Southgate J. (2014). A novel bidirectional positive-feedback loop between Wnt-β-catenin and EGFR-ERK plays a role in context-specific modulation of epithelial tissue regeneration. J. Cell Sci..

[44] Chen J., Long F. (2013). Beta-catenin promotes bone formation and suppresses bone resorption in postnatal growing mice. J. Bone Miner. Res..

[45] Hill T.P., Später D., Taketo M.M., Birchmeier W. (2005). Canonical Wnt/β-catenin signaling prevents osteoblasts from differentiating into chondrocytes. Dev. Cell.

[46] Holmen S.L., Zylstra C.R., Mukherjee A., Sigler R.E., Faugere M.C., Bouxsein M.L., Deng L., Clemens T.L., Williams B.O. (2005). Essential role of β-catenin in postnatal bone acquisition. J. Biol. Chem..

[47] Kulkarni N.H., Onyia J.E., Zeng Q., Tian X., Liu M., Halladay D.L., Frolik C.A., Engler T., Wei T., Kriauciunas A., Martin T.J., Sato M., Bryant H.U., Ma Y.L. (2006). Orally bioavailable GSK-3alpha/beta dual inhibitor increases markers of cellular differentiation in vitro and bone mass in vivo. J. Bone Miner. Res..

[48] Krause U., Harris S., Green A., Ylostalo J., Zeitouni S., Lee N., Gregory C.A. (2010). Pharmaceutical modulation of canonical Wnt signaling in multipotent stromal cells for improved osteoinductive therapy. Proc. Natl. Acad. Sci..

[49] Kim C.H., Neiswender H., Baik E.J., Xiong W.C., Mei L. (2008). β-catenin interacts with MyoD and regulates its transcription activity. Mol. Cell. Biol..

[50] Palmer H.G., Gonzalez-Sancho J.M., Espada J., Berciano M.T., Puig I., Baulida J., Quintanilla M., Cano A., de Herreros A.G., Lafarga M., Munoz A. (2001). Vitamin D(3) promotes the differentiation of colon carcinoma cells by the induction of E-cadherin and the inhibition of β-catenin signaling. J. Cell Biol..

[51] Shah S., Islam N., Dakshanamurthy S., Rizvi I., Rao M., Herrell R., Zinser G., Valrance M., Aranda A., Moras D., Norman A., Welsh J., Byers S.W. (2006). The molecular basis of vitamin D receptor and beta-catenin crossregulation. Mol. Cell.

