# Supplementary material for: Epidermal growth factor can signal via β-catenin to control proliferation of mesenchymal stem cells independently of canonical Wnt signalling
Source: Cell Signal. 2019 Jan;53:256–68. doi: 10.1016/j.cellsig.2018.09.021 (PMC6293317; doi:10.1016/j.cellsig.2018.09.021)
Supplement: Table S1 — Classical EGF and EGF/β-catenin signalling pathways significantly enrich different gene sets in MSCs. Lists of most significantly differentially enriched Hallmarks for classical EGF and EGF/β-catenin signalling pathways (upper panels). Lists of most significantly differentially enriched Biological Processes for classical EGF and EGF/β-catenin signalling pathways (lower panels). [file mmc1.docx]

**Table S1**

**Hallmarks:**

**Classical EGF**

| **Gene Set Name** | **Genes in Gene Set (K)** | **Genes in**  **Overlap (k)** | **k/K** | **p-value** | **FDR q-value** |
| --- | --- | --- | --- | --- | --- |
| E2F TARGETS | 200 | 47 | 0.235 | 4.95E-37 | 1.24E-35 |
| EPITHELIAL MESENCHYMAL TRANSITION | 200 | 47 | 0.235 | 4.95E-37 | 1.24E-35 |
| G2M CHECKPOINT | 200 | 44 | 0.22 | 2.03E-33 | 3.39E-32 |
| MITOTIC SPINDLE | 200 | 38 | 0.19 | 1.51E-26 | 1.88E-25 |
| MTORC1 SIGNALLING | 200 | 34 | 0.17 | 2.96E-22 | 2.96E-21 |
| TNFα SIGNALLING VIA NF-κB | 200 | 28 | 0.14 | 2.73E-16 | 2.28E-15 |
| ESTROGEN RESPONSE LATE | 200 | 26 | 0.13 | 1.92E-14 | 1.37E-13 |
| HYPOXIA | 200 | 25 | 0.125 | 1.51E-13 | 8.40E-13 |
| IL2 STAT5 SIGNALLING | 200 | 25 | 0.125 | 1.51E-13 | 8.40E-13 |
| UV RESPONSE DN | 144 | 21 | 0.1458 | 6.36E-13 | 3.18E-12 |

**Hallmarks:**

**EGF/β-catenin**

| **Gene Set Name** | **Genes in Gene Set (K)** | **Genes in Overlap (k)** | **k/K** | **p-value** | **FDR q-value** |
| --- | --- | --- | --- | --- | --- |
| HYPOXIA | 200 | 7 | 0.035 | 3.22E-06 | 8.05E-05 |
| INFLAMMATORY RESPONSE | 200 | 7 | 0.035 | 3.22E-06 | 8.05E-05 |
| COAGULATION | 138 | 5 | 0.0362 | 7.31E-05 | 1.22E-03 |
| ANDROGEN RESPONSE | 101 | 4 | 0.0396 | 2.83E-04 | 2.93E-03 |
| EPITHELIAL MESENCHYMAL TRANSITION | 200 | 5 | 0.025 | 4.11E-04 | 2.93E-03 |
| IL2 STAT5 SIGNALLING | 200 | 5 | 0.025 | 4.11E-04 | 2.93E-03 |
| KRAS SIGNALLING UP | 200 | 5 | 0.025 | 4.11E-04 | 2.93E-03 |
| UV RESPONSE DN | 144 | 4 | 0.0278 | 1.07E-03 | 6.71E-03 |
| APOPTOSIS | 161 | 4 | 0.0248 | 1.62E-03 | 8.99E-03 |
| INTERFERON ALPHA RESPONSE | 97 | 3 | 0.0309 | 3.44E-03 | 1.48E-02 |

**Table S1 continued**

**GO: Biological Process**

**Classical EGF**

| **GO Term** | **Genes in Gene Set (K)** | **Genes in Overlap (k)** | **k/K** | **p-value** | **FDR q-value** |
| --- | --- | --- | --- | --- | --- |
| MOVEMENT OF CELL OR SUBCELLULAR COMPONENT | 1275 | 122 | 0.0957 | 3.25E-49 | 1.44E-45 |
| TISSUE DEVELOPMENT | 1518 | 129 | 0.085 | 2.24E-46 | 4.97E-43 |
| REGULATION OF PHOSPHORUS METABOLIC PROCESS | 1618 | 132 | 0.0816 | 1.64E-45 | 2.43E-42 |
| CELL CYCLE | 1316 | 118 | 0.0897 | 1.08E-44 | 1.20E-41 |
| REGULATION OF ORGANELLE ORGANIZATION | 1178 | 108 | 0.0917 | 9.20E-42 | 8.16E-39 |
| CELL DEVELOPMENT | 1426 | 118 | 0.0827 | 3.32E-41 | 2.46E-38 |
| REGULATION OF CELL DEATH | 1472 | 119 | 0.0808 | 1.49E-40 | 8.44E-38 |
| POSITIVE REGULATION OF RESPONSE TO STIMULUS | 1929 | 137 | 0.071 | 1.58E-40 | 8.44E-38 |
| REGULATION OF MULTICELLULAR ORGANISMAL DEVELOPMENT | 1672 | 127 | 0.076 | 1.71E-40 | 8.44E-38 |
| POSITIVE REGULATION OF CELLULAR COMPONENT ORGANIZATION | 1152 | 105 | 0.0911 | 2.28E-40 | 1.01E-37 |

**GO: Biological Process**

**EGF/β-catenin**

| **GO Term** | **Genes in Gene Set (K)** | **Genes in Overlap (k)** | **k/K** | **p-value** | **FDR q-value** |
| --- | --- | --- | --- | --- | --- |
| RESPONSE TO EXTERNAL STIMULUS | 1821 | 31 | 0.017 | 7.01E-15 | 3.11E-11 |
| CELLULAR RESPONSE TO ORGANIC SUBSTANCE | 1848 | 30 | 0.0162 | 7.02E-14 | 1.56E-10 |
| CELLULAR RESPONSE TO STRESS | 1565 | 25 | 0.016 | 1.60E-11 | 2.36E-08 |
| DEFENSE RESPONSE | 1231 | 22 | 0.0179 | 3.65E-11 | 3.31E-08 |
| NEGATIVE REGULATION OF RESPONSE TO STIMULUS | 1360 | 23 | 0.0169 | 3.73E-11 | 3.31E-08 |
| RESPONSE TO OXYGEN CONTAINING COMPOUND | 1381 | 23 | 0.0167 | 5.04E-11 | 3.73E-08 |
| POSITIVE REGULATION OF CELL COMMUNICATION | 1532 | 24 | 0.0157 | 6.36E-11 | 4.03E-08 |
| INTRACELLULAR SIGNAL TRANSDUCTION | 1572 | 24 | 0.0153 | 1.07E-10 | 5.95E-08 |
| POSITIVE REGULATION OF MOLECULAR FUNCTION | 1791 | 25 | 0.014 | 2.69E-10 | 1.27E-07 |
| RESPONSE TO WOUNDING | 563 | 15 | 0.0266 | 2.87E-10 | 1.27E-07 |
