# Supplementary material for: Epidermal growth factor can signal via β-catenin to control proliferation of mesenchymal stem cells independently of canonical Wnt signalling
Source: Cell Signal. 2019 Jan;53:256–68. doi: 10.1016/j.cellsig.2018.09.021 (PMC6293317; doi:10.1016/j.cellsig.2018.09.021)
Supplement: Supplementary file 2 — Supplementary material [file mmc2.docx]

**Supplemental Material**

**Western blot analysis**

| **Primary Antibody Target** | **Dilution** | **Supplier** |
| --- | --- | --- |
| Active β-catenin | 1:2000 | Merck |
| pERK | 1:2000 | R and D Systems |
| pc-Jun | 1:1000 | Abcam |
| GAPDH | 1:2000 | Gene Tex |
| **Secondary Antibody Target** | **Dilution** | **Supplier** |
| Mouse IgG (HRP) | 1:2000 | Santa Cruz Biotechnology |
| Rabbit IgG (HRP) | 1:2000 | Santa Cruz Biotechnology |

Antibodies used for western blot analysis

**Quantitative real time polymerase chain reaction (QPCR)**

| **Gene** | **Forward Primer Sequence** | **Reverse Primer Sequence** |
| --- | --- | --- |
| Axin2 | TTATGCTTTGCACTACGTCCCTCCA | CGCAACATGGTCAACCCTCAGAC |
| c-Myc | TCAAGAGGCGAACACACAAC | GGCCTTTTCATTGTTTTCCA |
| LEF1 | CAGGAGCCCTACCACGACAA | TTGTAGAGGCCTCCATCTGGAT |
| RPS27a | TGGATGAGAATGGCAAAATTAGTC | CACCCCAGCACCACATTCA |

Primer sequences used for Quantitative real time polymerase chain reaction (QPCR)

**Statistical Analysis**

| **Experiment** | **Figure** | **Statistical Test** | **Significance** |
| --- | --- | --- | --- |
| MTT assay of viable cell numbers (hTERT MSCs) | Figure 1C  (except Y102 at 24 hours) | One way ANOVA and Holm-Sidak | *p<0.05 **p<0.005  ***p<0.001 |
| MTT assay of viable cell numbers (hTERT MSCs) | Figure 1C  (Y102 at 24 hours) | Kruskal Wallis | *p<0.05 **p<0.005  ***p<0.001 |
| TCF reporter assays  (C_3_H_10_T_1/2_) | Figure 3D  (M50 samples relative to control) | One way ANOVA and Holm-Sidak | ***p<0.001 |
| Design of Experiments (DoE)-based analysis of combined EGF/Wnt effects (hTERT MSCs) | Figure 4D | ANOVA | p≤0.05 |
| MTT assay of viable cell numbers (MSCs) | Figure 5A | Ranked ANOVA Kruskal Wallis | *p<0.05 |
| MTT assay of viable cell numbers (MSCs) | Figure 5B  (120 hour data - Donor 1) | Ranked ANOVA Kruskal Wallis | *p<0.05 |
| MTT assay of viable cell numbers (MSCs) | Figure 5B  (120 hour data - Donor 2) | One way ANOVA and Holm-Sidak | *p<0.05  **p<0.005 ***p<0.001 |
| Edu Assay of Cell Proliferation | Figure 5C | One way ANOVA with Tukey’s multiple comparison test | *p<0.05  **p<0.01  ***p<0.001  ****p<0.0001 |
| MTT assay of viable cell numbers (MSCs) | Figure S4C  (120 hour data - Donor 1) | Ranked ANOVA Kruskal Wallis | *p<0.05 |
| MTT assay of viable cell numbers (MSCs) | Figure S4C  (120 hour data - Donor 2) | One way ANOVA | *p<0.05  **p<0.005 ***p<0.001 |

Statistical methods used to analyse experimental data
